# Early X chromosome inactivation during human preimplantation development revealed by single-cell RNA-sequencing

**DOI:** 10.1038/s41598-017-11044-z

**Published:** 2017-09-07

**Authors:** Joana C. Moreira de Mello, Gustavo R. Fernandes, Maria D. Vibranovski, Lygia V. Pereira

**Affiliations:** 1 National Laboratory for Embryonic Stem Cells (LaNCE), Department of Genetics and Evolutionary Biology, Institute of Biosciences, University of São Paulo, SP 05508-090 São Paulo, Brazil; 2 Laboratory of Evolutionary Genomics, Department of Genetics and Evolutionary Biology, Institute of Biosciences, University of São Paulo, SP 05508-090 São Paulo, Brazil

## Abstract

In female mammals, one X chromosome is transcriptionally inactivated (XCI), leading to dosage compensation between sexes, fundamental for embryo viability. A previous study using single-cell RNA-sequencing (scRNA-seq) data proposed that female human preimplantation embryos achieve dosage compensation by downregulating both Xs, a phenomenon named dampening of X expression. Using a novel pipeline on those data, we identified a decrease in the proportion of biallelically expressed X-linked genes during development, consistent with XCI. Moreover, we show that while the expression sum of biallelically expressed X-linked genes decreases with embryonic development, their median expression remains constant, rejecting the hypothesis of X dampening. In addition, analyses of a different dataset of scRNA-seq suggest the appearance of X-linked monoallelic expression by the late blastocyst stage in females, another hallmark of initiation of XCI. Finally, we addressed the issue of dosage compensation between the single active X and autosomes in males and females for the first time during human preimplantation development, showing emergence of X to autosome dosage compensation by the upregulation of the active X chromosome in both male and female embryonic stem cells. Our results show compelling evidence of an early process of X chromosome inactivation during human preimplantation development.

## Introduction

In eutherian mammals, X-linked gene dosage compensation between females and males is achieved by the transcriptional inactivation of one X in female cells (XCI) during embryonic development^1^. In mice, the process starts at 2- to 4-cell stage female embryos, where the long non-coding *Xist* RNA is first observed coating the paternal X *in cis* leading to imprinted XCI. At the blastocyst stage, cells from the epiblast reactivate the paternal X, and random XCI subsequently takes place, resulting in random inactivation in the embryo proper and imprinted XCI in mouse placenta^2, 3^.

In contrast, the dynamics of XCI during human embryonic development is still much unknown, with reports presenting conflicting results^4–6^. While accumulation of *XIST* RNA in one X exclusively in female embryos was shown at the morula and blastocyst stage, along with inactivation of one gene in the *XIST*-coated region in blastocysts^4^, another report employing equivalent *in situ* analysis detected *XIST* RNA accumulation in both Xs of female and in the single X of male morulas and blastocysts, but no transcriptional silencing of three X-linked genes^5^, arguing against XCI during human preimplantation development. These conflicting results may be due to limitations of *in situ* methods and to the small number of X-linked genes assayed, but they indicate important differences in XCI between mouse and humans.

More recently, using single-cell RNA-sequencing (scRNA-seq) from human preimplantation embryos, Petropoulos *et al*.^7^ showed that dosage compensation between males and females is achieved by day 7 (E7) blastocysts. However, since no sign of monoallelic transcriptional silencing of X-linked genes was observed, the authors concluded that, instead of XCI, dosage compensation during human preimplantation development occurs by downregulation of both Xs in females, a phenomenon named *dampening* of X expression.

Here, we used a novel pipeline to analyze XCI in human preimplantation embryos using data from Petropoulos *et al*.^7^ (embryos from 8-cell to the E7 blastocyst – dataset-1); from previous scRNA-seq study of human preimplantation embryos^8^ (from oocytes to the E6 blastocyst stage, and male human embryonic stem cells – dataset-2), and of female adult fibroblast^9^; and from RNA-seq of whole blastocysts^10^ and female human embryonic stem cells (hESCs, H9 cell line)^11^, Table 1. Starting from oocytes, we give a complete panorama of dosage compensation during the initial stages of embryo development, showing decrease of biallelic and concomitant increase of monoallelic X-linked gene expression along development, consistent with random XCI in human preimplantation embryos rather than dampening of X expression. In addition, we address for the first time the issue of dosage compensation between the active X (X_a_) and autosomes during human embryonic development, and show that primed hESCs have an upregulated X_a_.

**Table 1:** Number of embryos and cells analyzed in each dataset.

Stage	Embryonic day		Dataset #^17^		Dataset #^28^	
			Embryo	cell	Embryo	cell
Oocyte			—	—	3	3
Zygote			—	—	3	3
2-cell			—	—	3	6
4-cell			—	—	3	12
8-cell	(E3)	Male	6	40	1	8
		Female	4	25	2	12
Morula	(E4)	Male	7	97	2	12
		Female	7	88	—	—
Blastocyst	(E5)	Male	13	204	—	—
		Female	9	160	—	—
Blastocyst	(E6)	Male	6	119	2	22
		Female	10	234	1	8
Blastocyst	(E7)	Male	7	175	—	—
		Female	10	290	—	—

## Results

### Initiation of the X inactivation process in human development

Single-cell RNA-seq data were screened for informative SNPs, generating an RNA-based genotype of each embryo and cell line. While Petropoulos *et al*.^7^ used a minimum of 3 reads as SNP call threshold, possibly generating a high rate of false positive and negative calls of biallelic expression, our strategy combined a more stringent threshold for SNP calling (number of reads ≥20 for SNP call), and the use of several filters to avoid artifacts (see Methods).

To determine the fraction of X-linked mono and biallelically expressed genes per cell in each embryo, we looked for informative transcribed SNPs outside of the pseudoautosomal region. To consider a gene as informative in an embryo, it should either show biallelic expression in at least one cell; or have at least two cells of the same embryo expressing just one, but different, alleles (*e.g. A* and *A’*), which was considered monoallelic expression. Therefore, an imprinted X-linked gene, which would express the same allele (*e.g. A’*) in all cells of an embryo, would be considered a non-informative gene rather than monoallelic. Thus, imprinted XCI would lead to no informative monoallelically expressed X-linked genes in latter stage female embryos (similar to male embryos, Supplementary Fig. S1). Importantly, to avoid a bias in the number of informative SNPs in embryos with more sequenced cells, leading to a false increase in monoallelically expressed genes, we randomly picked four cells of each sample for monoallelic expression analyses (similar results were obtained using all cells from each embryo - Supplementary Fig. S1).

**Figure 1:**
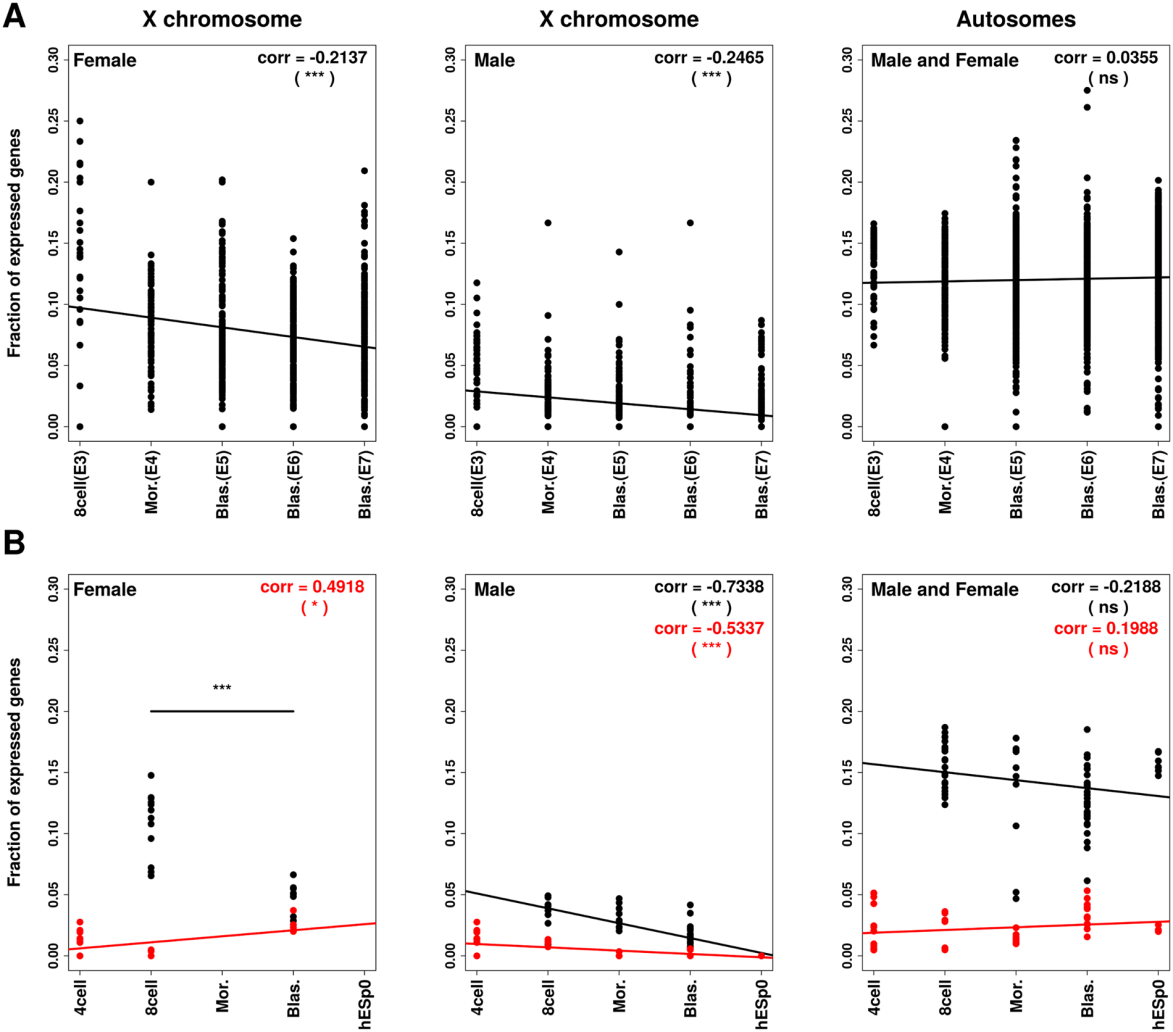
Allelic expression during preimplantation development. Analyses of changes in fractions of biallelically and monoallelically expressed genes during development in male and female embryos. Fractions of biallelically (black) and monoallelically (red) expressed genes per total of expressed genes in each cell for each developmental stage are shown. (**A**) Dataset-1; (**B**) Dataset-2. Pearson’s r values (corr) are depicted in each panel. Non-paired Wilcoxon test was performed to compared the fractions of biallelically expressed X-linked genes between two stages in dataset-2. *P*-value (*) < 0.05; (***) ≤0.001; (ns) not significant. Numbers of genes analyzed and similar analysis of individual autosomes and are shown in Supplementary Fig. S3 and Supplementary Fig. S4, respectively.

Exploring dataset-1, we found consistent low coverage of aligned sequences to genes, SNP calls and heterozygous gene detection that affect analysis of allele-specific gene expression (Supplementary Fig. S2). Nevertheless, we were able to find a significant decrease in the fraction of X-linked biallelically expressed genes in female embryos from 8-cell to E7 blastocyst stage (Pearson’s r = −0.2137; *P*-value = 1.538 × 10^−9^), consistent with XCI (Fig. 1A, left panel). This decrease cannot be attributed to lower sequencing depth of latter staged embryos (Supplementary Fig. S2). Male embryos showed decreased biallelic expression of X-linked gene consistent with degradation of maternal RNA (Fig. 1A, middle panel), while, in contrast, no significant negative correlation was found for autosomal genes (Fig. 1A, right panel). When analyzed individually, only 2 out of 22 autosomes in females showed significant decrease in biallelic expression (Supplementary Fig. S4).

In order to further investigate XCI, we performed the same analysis in a different data set of scRNA-seq from human preimplantation embryos^8^, spanning from oocytes to E6 blastocyst stage (dataset-2) (Fig. 1B). Embryos were sexed based on expression of Y-linked genes, using oocytes and male human embryonic stem cells at passage 0 (hESp0) as female and male controls, respectively. We identified two 8-cell embryos and one blastocyst as female; and one 8-cell embryo, two morulas, and two blastocysts as male (Supplementary Fig. S5 and Supplementary Table S1). Sexing zygotes, 2- and 4-cell stage embryos was not possible because they have not undergone full embryonic genome activation (EGA)^8, 12, 13^.

Analysis of *XIST* expression in single cells from dataset-2 shows that at the 8-cell and blastocyst stages *XIST* expression in female was significantly higher than in pre-EGA or male embryos (Supplementary Fig. S6, Supplementary Table S2). Since no informative SNP in *XIST* was found, we could not distinguish mono from biallelic *XIST* expression using dataset-2 (Supplementary Note, Supplementary Fig. S6).

To test whether the process of X-linked gene silencing had initiated in female embryos, we analyzed allele-specific gene expression in scRNA-seq dataset-2. Four aneuploid cells in male morula #2 were excluded from subsequent analyses (see Methods, Supplementary Fig. S7 and Supplementary Table S3). We found that the mean number of monoallelically and biallelically expressed X-linked genes per cell was 25 and 2 times higher, respectively, than those found in dataset-1 (Supplementary Table S3). As in dataset-1, the number of reads aligned to genes in dataset-2 was not lower in latter stage embryos (Supplementary Fig. S2).

In female embryos of dataset-2, the proportion of X-linked genes biallelically expressed decreased with development (Non-paired Wilcoxon test *P*-value = 3.175 × 10^−5^) (Fig. 1B, left panel), corroborating the results we obtained from the scRNA-seq dataset-1 (Fig. 1A). However, female, but not male, embryos showed a significant decrease also in the proportion of biallelically expressed autosomal genes (Supplementary Fig. S4).

Due to the greater coverage of this set of scRNA-seq (Supplementary Fig. S2), we were able to analyze monoallelic expression from the X chromosome. We detected a significant positive correlation between the fractions of monoallelically expressed X-linked genes and the developmental stage in female embryos (Pearson’s r = 0.4918; *P*-value = 0.0146), indicating an increase in monoallelic expression from the X consistent with random XCI (Fig. 1B, left panel; see Fig. S4B for Bonferroni’s correction when considering all chromosomes analyzed separately). In contrast, no significant correlation between monoallelic expression of autosomal genes and development was seen in male or female embryos (Fig. 1B, right panel).

**Figure 2:**
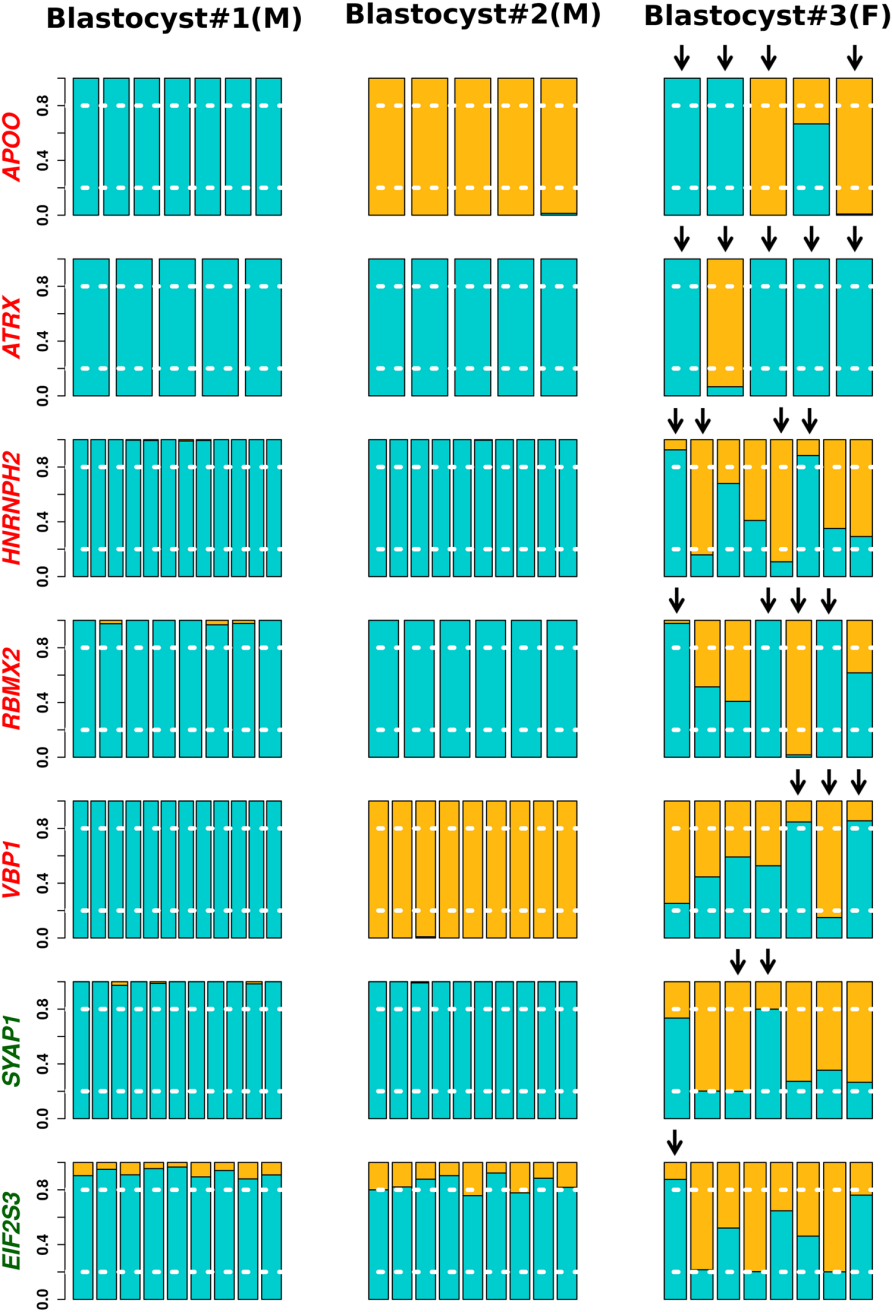
Allele-specific expression of X-linked genes at the blastocyst stage. Cells are grouped by embryo where each bar represents a single cell. Allelic expression pattern was detected by informative SNPs in the female blastocyst #3. M, male; F, female. Allelic relative expression ratios ≤0.2 or ≥0.8 were considered as monoallelic expression (white dotted line). Relative expression of reference and alternative alleles in blue and orange, respectively. Arrows point to cells with random XCI, *i.e*., cells from the same embryo presenting monoallelic expression from different alleles. Y axis: relative expression ratio. Genes in red and green were described as subjected to and escaping XCI, respectively^36^. Expression pattern of gene *APOO* detected by rs8680; *ATRX*, rs3088074; *HNRNPH2*, rs41307260; *RBMX2*, rs142885112; *VBP1*, rs11887; *SYAP1*, rs144608858 and *EIF2S3*, rs5949273. All blastocysts from dataset-2.

Figure 2 depicts graphically the expression pattern of seven X-linked genes in dataset-2 E6 blastocysts. Although some female cells still exhibit biallelic expression, a considerable proportion of them already present monoallelic expression, a signature of random XCI. As expected, male cells express predominantly the same allele reflecting the single X chromosome (Fig. 2). The same analysis with informative genes common among female 8-cell embryos and blastocyst indicates initiation of the transition from biallelic to monoallelic expression (Supplementary Fig. S8).

### No evidence of dampening of X expression

**Figure 3:**
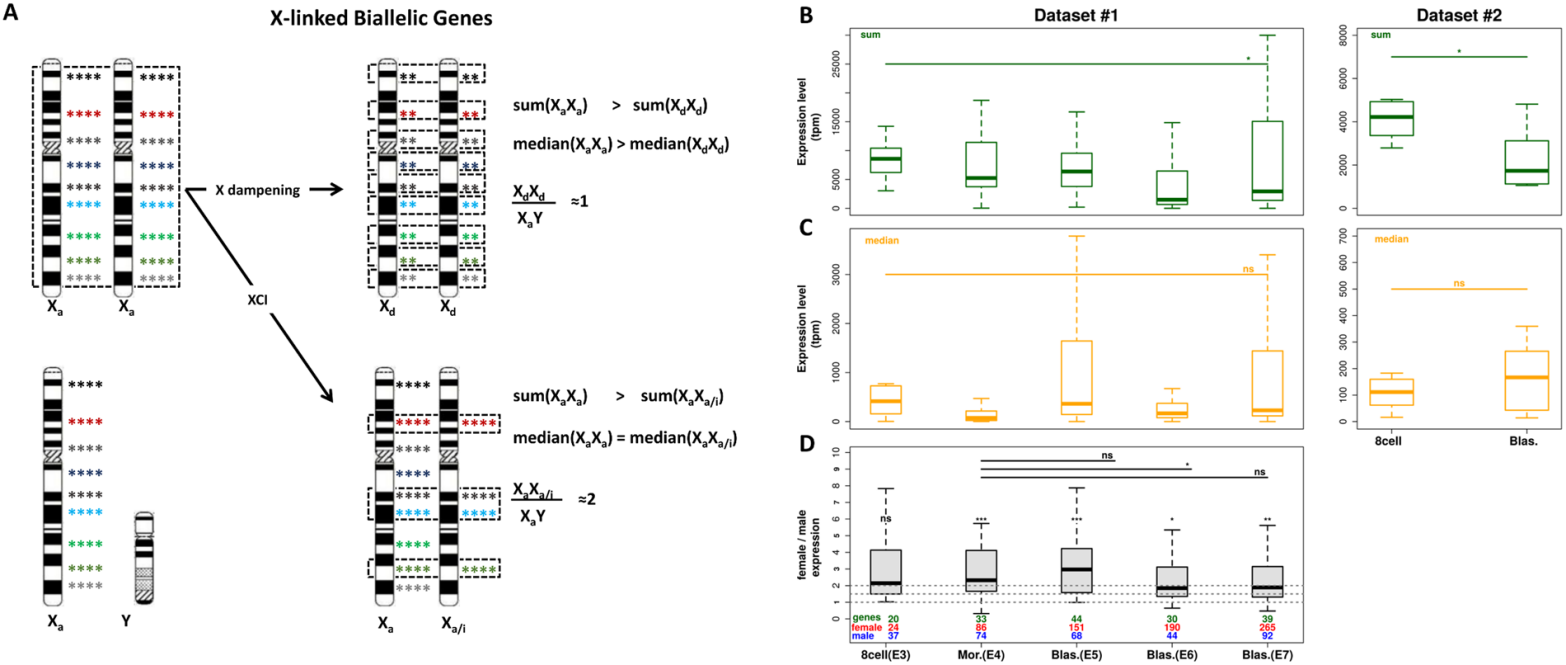
Expression of biallelically expressed X-linked genes during female development. (**A**) Predictions of sum, median and female to male ratio of expression of biallelically expressed X-linked genes for X dampening and XCI hypotheses. Colored asterisks represent expression levels of X-linked genes. Dotted lines highlight biallelically expressed genes. X_d_: dampened X; X_a/i_: X chromosome undergoing XCI. X dampening would lead to decreased expression levels of both alleles, while XCI to a decrease in the number of biallelically expressed genes. Both lead to a decrease in the expression sum of biallelically expressed genes (sum). However, the median expression of biallelically expressed genes (median) should decrease with X dampening and not change with XCI. The female to male ratio of genes biallelically expressed in females should approach 1 in the case of X dampening and 2 in the case of XCI; (**B**) Distributions of sums (green) and (**C**) medians (orange) of biallelically expressed X-linked genes in each developmental stage for dataset-1 and -2. (**D**) Distributions of female to male ratio of expression of X-linked genes biallelically expressed in females (dataset-1). Numbers of genes (green), of female (red) and of male (blue) cells analyzed are indicated for each stage. Differences between males and females in each stage are indicated above the respective boxplots. Differences in female to male ratios between stages are shown. Wilcoxon tests: *P*-value (*) < 0.05; (**) ≤0.01; (***) ≤0.001; (ns) not significant. Expression levels differ between females and males at morula and blastocysts stages. Despite the borderline difference between E4 and E6 (*P*-value = 0.045), there are no differences on female-to-male expression ratio among developmental phases, indicating no down-regulation of female X-linked biallelically expressed genes.

We then looked at global expression level of biallelically expressed X-linked genes during development in scRNA-seq datasets-1 and -2 (Fig. 3). In both datasets we observed a decrease in the sum of expression levels of biallelically expressed X-linked genes, which could be due to dampening (decrease in the expression level of each biallelically expressed gene) or to XCI (decrease in the number of genes biallelically expressed) (Fig. 3A and B). To discern between the two mechanisms, we looked at the median of the expression of biallelically expressed X-linked genes, which, in the case of dampening, should also decrease during development (Fig. 3A). As shown in Fig. 3C, although presenting considerable variation among cells, in both datasets the median of the expression levels of biallelically expressed X-linked genes does not decrease during development.

To corroborate these results, we analyzed the female-to-male relative expression level of each biallelically expressed X-linked gene for each developmental stage from E3 to E7. X dampening would lead to a ratio of biallelically expressed genes between females and males close to 1, while in the case of XCI this ratio would be around 2 (only genes that have not undergone XCI have biallelic expression) (Fig. 3A). Figure 3D shows that in E6 and E7 the expression level of biallelically expressed X-lined genes in females is on average two times higher than in males, again arguing against X dampening. Moreover, female-to-male relative expression levels of biallelically expressed X-linked genes do not change from E4 to E7. Therefore, we conclude that, rather than the expression levels, it is the number of genes biallelically expressed that diminish in females from the 8-cell to the blastocyst stage, consistent with an ongoing establishment of XCI (see also Fig. 1).

### X-linked dosage compensation between males and females

**Figure 4:**
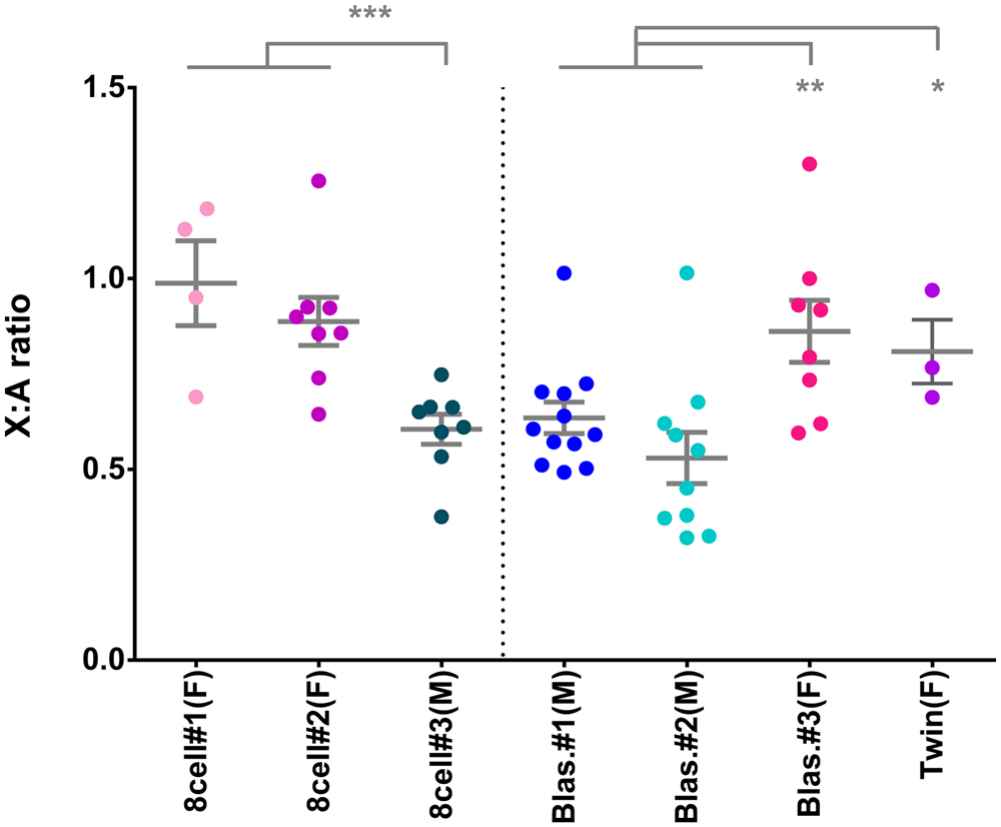
X chromosome dosage compensation. Scatter plots of the X:A expression ratio (mean ± s.e.m. in gray) in single cells of 8-cell embryos and blastocysts from dataset-2; Twin corresponds to ICMs and trophectoderm of a female blastocyst with two ICMs^10^. *P*-values: (*) <0.05, (**) ≤0.01, (***) ≤0.001 non-paired Wilcoxon test.

In order to evaluate whether the increase of X-linked monoallelic expression was accompanied by dosage compensation, we compared the ratio of X to autosomal gene expression levels (X:A) in male and female embryos from dataset-2, which should be equivalent once XCI is complete (Fig. 4). Female 8-cell stage embryos #1 and #2 showed similar X:A ratios (mean 0.988 and 0.887, respectively), while male 8-cell embryo #3 had a significantly lower X:A ratio (mean 0.605; Wilcoxon test, *P*-value = 0.0004), consistent with lack of dosage compensation. Likewise, female blastocyst #3 and twin ICMs and trophectoderm showed a significantly higher X:A ratio when compared to male blastocysts #1 and #2 (non-paired Wilcoxon test, *P*-value 0.003018, Fig. 4). Thus, our data show that, despite *XIST* expression (Supplementary Fig. S6) and increase in X-linked monoallelically expressed genes (Fig. 1B, left panel), the latter is still not sufficient to lead to detectable dosage compensation of the X chromosome between males and females by the E6 blastocyst stage (Fig. 4 and Supplementary Fig. S1).

Looking at the total sum of X chromosome rpkm during development, Petropoulos *et al*.^7^ detected equivalent X-linked expression levels in male and female embryos only at the E7 blastocyst stage. Analyzing their scRNA-seq data (dataset-1), we observed similar X:A ratios in male and female embryos only at E7 blastocysts (Supplementary Fig. S9), corroborating that dosage compensation in humans is achieved at that latter embryonic stage. Taken together, the analyses of both datasets indicate that in humans random XCI progresses through early embryonic development, leading to dosage compensation just before implantation.

### X-Autosomes dosage compensation

**Figure 5:**
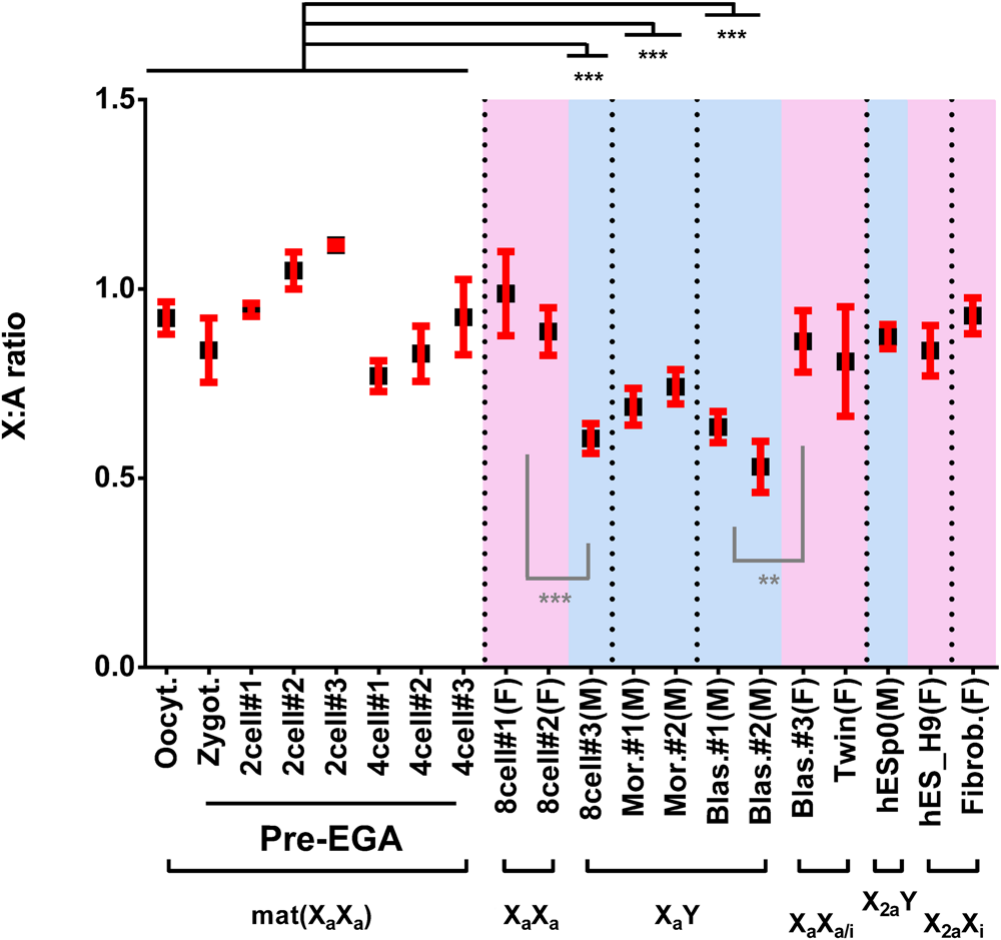
X-autosomes dosage compensation during development. Mean X:A expression ratio per embryo/sample (mean ± s.e.m. in red)*. P*-values: (**) ≤0.01, (***) ≤0.001 non-paired Wilcoxon test; sex of post-EGA stages is indicated and highlighted by background color: females (F, pink), males (M, blue). Embryos and male hESC (hESCp0) from dataset-2; twin corresponds to ICMs and trophectoderm of a female blastocyst with two ICMs^10^; Fibrob. is a female fibroblast cell line^9^; H9, female hESC line^11^. See Supplementary Table S4, for single-cell X:A expression ratios. Below, the activity of the X chromosomes in the respective embryos/cells. mat(X_a_X_a_): maternal mRNA from primordial germ cells with two active X chromosomes; X_a/i_: X chromosome undergoing XCI; X_2a_: upregulated active X.

Interestingly, our analyses of the female fibroblast cell line, which has undergone complete random XCI^9^, showed an X:A ratio close to 1, similar to those of the pre-XCI female embryos with two X_a_’s (Fig. 5). This is consistent with dosage compensation between the X chromosome and autosomes achieved by transcriptional upregulation of the single X_a_ in adult cells, as proposed by Ohno^14^. In addition, the X:A ratio in the male hESp0 cells was not different from that in female fibroblasts, but significantly higher than in male blastocysts (Fig. 5, *P*-value = 0.0005 non-paired Wilcoxon test); and the H9 female hESCs line, which has undergone XCI^11, 15^, also presented an X:A ratio close to 1 and equivalent to pre-XCI female embryos with two X_a_ (Fig. 5). These results show that upregulation of the human X_a_ does not take place by the E6 blastocyst stage, but can be detected in male and female pluripotent cells *in vitro*. Together with the observation of X-linked dosage compensation at E7^7^, these results indicate intense epigenetic modifications of the X chromosomes during this narrow window of development. Finally, pre-EGA embryos also displayed X:A ratios similar to female pre-XCI embryos (Fig. 5). Since RNAs present in pre-EGA embryos are those transcribed from maternal cells with two X_a_, these data suggest that, in addition to reactivating the inactive X (X_i_)^16^, oogenesis includes erasing X_a_ upregulation.

## Discussion

Single-cell RNA-seq technology has allowed more detailed analysis of gene expression during human preimplantation development, leading to better characterization of lineage segregation and of the *in vivo* pluripotent state^7, 8^. In this study, we used those data to investigate the process of XCI. By globally analyzing X chromosome expression during human embryonic development (dataset-1^7^ and dataset-2^8^), we were able to detect decrease of biallelic and increase of monoallelic X-linked gene expression from the 4-cell to the blastocyst stage, indicating that, contrary to the findings originally published for dataset-1^7^, initiation of XCI does take place during human preimplantation development.

Analyzing scRNA-seq from dataset-1, Petropoulos *et al*.^7^ found no evidence of XCI, despite observing dosage compensation between male and female E7 blastocysts. Therefore, they proposed that at that preimplantation stage dosage compensation was achieved by expression dampening of both X’s in the female, albeit not formally testing this hypothesis. Indeed, due to the relatively low coverage, we did not have enough statistical power to rigorously analyze allele-specific gene expression in their data. Nevertheless, we could show decrease of the fraction of biallelically expressed X-linked genes with development, indicative of XCI.

In addition to showing indications of an ongoing process of XCI, we present compelling evidence against X dampening in human preimplantation embryos. Specifically, we found no decrease in the median expression of biallelic X-linked genes from the 8-cell to the blastocyst stage in females, which would be expected if dosage compensation were due to dampening of X-linked expression in both chromosomes. Rather, we show that the lower expression sum of biallelically expressed X-linked genes in latter stage embryos results from the decrease in their number, another hallmark of XCI. Finally, we show that at the E7 blastocyst stage, expression of biallelically expressed X-linked genes is significantly higher in females than in males, again arguing against X dampening.

Our results reveal additional differences between human and murine preimplantation development. We place induction of *XIST* expression at the 8-cell stage, while in mice *Xist* expression is first detected at the 2- to 4-cell embryo in an imprinted pattern, both coinciding with the respective time of EGA^2, 3^. However, we show that in human males the maternal *XIST* allele is also expressed in preimplantation embryos, corroborating lack of imprinted XCI^17^. Moreover, using stringent statistical analysis, we detect progression of random XCI from the 8-cell to the E6 blastocyst stages in dataset-2. In contrast, mice undergo rapid random XCI only in the epiblast and upon differentiation^2, 3^.

Finally, we investigated the issue of X to autosomes dosage compensation for the first time during human embryonic development. It has been recently shown by scRNA-seq that upregulation of the murine X_a_ initiates at the 4-cell stage, leading to X:A dosage compensation by E4.5, with the onset of random XCI in females^18^. Our results are consistent with the establishment of human X:A dosage compensation after the E6 blastocyst stage, as shown *in vitro* in male and female embryonic stem cells and in female fibroblasts. Thus, in addition to inactivation of a single X in females, the human peri-implantation stage also involves upregulation of the X_a_ in both sexes. Finally, we show evidence that X_a_ upregulation is erased during oogenesis, reflected in the observed X:A dosage compensation of maternal RNAs in X_a_X_a_ oocytes and pre-EGA embryos.

Our observations help understand the differences between human and mouse blastocyst-derived pluripotent stem cells (PSCs) in regards to the epigenetic state of the X chromosome. While the murine cells model well the *in vivo* pre-XCI state and undergo random XCI upon differentiation, human PSCs show variable states of XCI (reviewed in ref. 19). However, specific culture conditions shift human PSCs into a naïve state, more similar to mouse PSCs, which includes the presence of two X_a_’s (reviewed in ref. 20). Nevertheless, our findings show that in order to recapitulate the epigenetic state of the X chromosome in the human epiblast, naïve human PSCs must be in the process of establishing XCI. Thus, to capture the human pre-XCI state and develop an *in vitro* model of initiation of XCI, conditions to culture morulas must be established.

Recently, X chromosome dampening has been reported in naïve hESCs, where the transition of primed lines of hESCs to the naïve state lead to reactivation of the X_i_ and an overall decrease in X-linked gene expression, albeit not sufficient to achieve dosage compensation^21, 22^. However, in their analysis of global X-linked expression levels, the authors did not take into account the possibility of X-upregulation, as we show in male and female primed hESC. Indeed, the unexpected decrease of X-linked gene expression in male naïve compared to male primed hESCs observed by Theunissen *et al*.^21^ may be due to erasure of upregulation of the X_a_ in the naïve cells. Therefore, the epigenetic remodeling of the X chromosomes in the transition from the primed to the naïve state is more complex than previously anticipated, and it should be reanalyzed in light of the findings reported in this work that primed male and female hESC have an upregulated X_a_.

**Figure 6:**
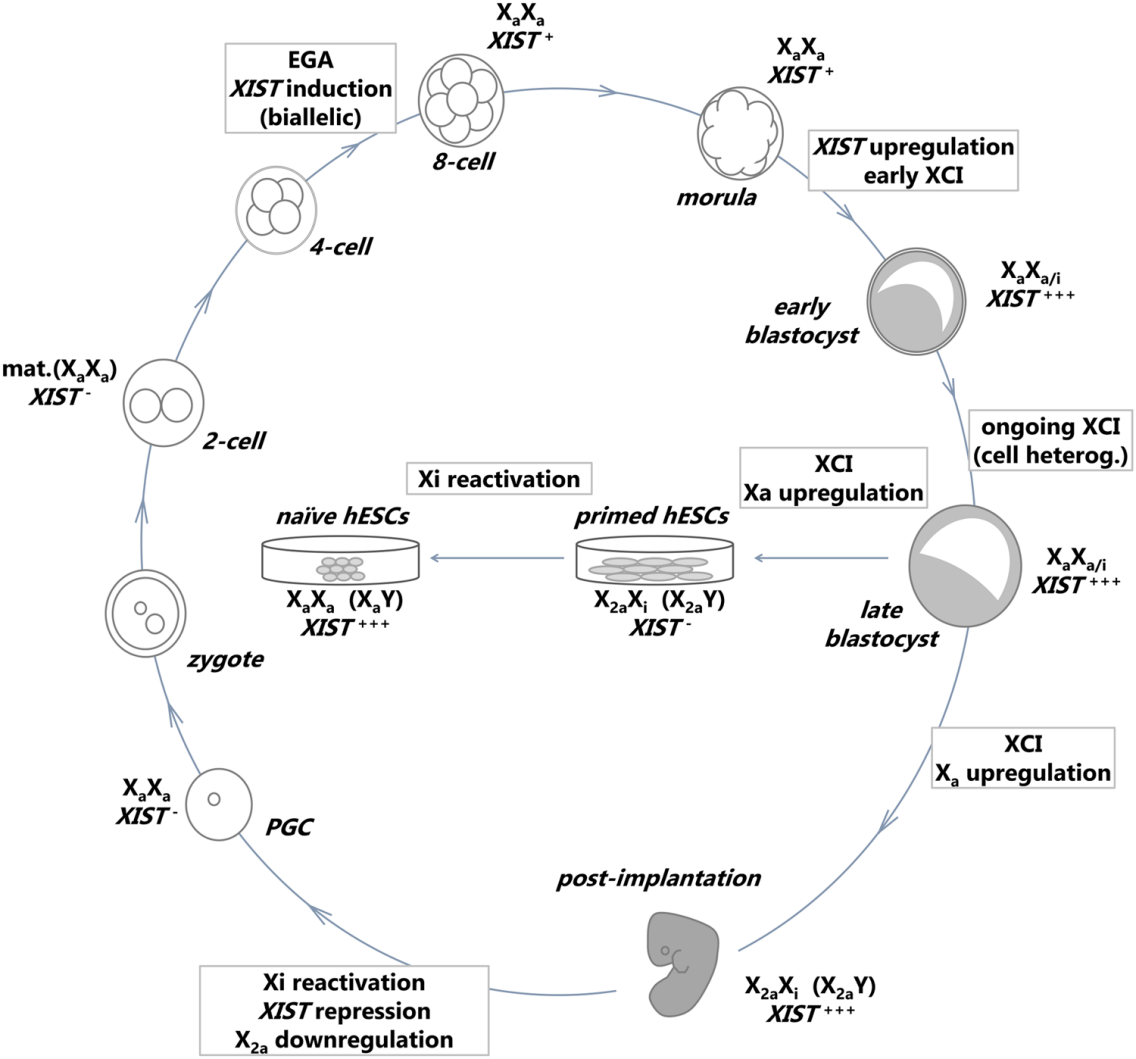
Model of X chromosome activity during female human embryonic development. *XIST* expression is induced biallelically at the 8-cell stage, concomitant with EGA^8, 12, 13^, and is upregulated in female early blastocysts, where early signs of XCI are detected (X_a_X_a/i_). XCI is mostly complete in late blastocyst (X_a_X_a/i_), approaching dosage compensation between male and female embryos^7^. Upon implantation, XCI is complete and the single X_a_ is upregulated, leading to X-autosome dosage compensation. Primordial germ cells (PGC) repress *XIST*, reactivate the X_i_^16^ and erase X_a_-upregulation, thus becoming X_a_X_a_. Therefore, pre-EGA embryos have maternal RNAs from X_a_X_a_/*XIST* ^−^ cells [mat.(X_a_X_a_)]. Primed hESCs derived from blastocysts have variable and unstable states of XCI^37^, but after many passages most of them undergo XCI, repress *XIST* and upregulate the X_a_ (male lines only upregulate the X_a_). Transition to the naïve state leads to *XIST* expression at levels equivalent to those of female blastocysts and monoallelic in most cells^15, 21, 22^, and loss of X_a_-upregulation in male and female cells. It remains to be further understood the mechanisms behind the decreased X-linked expression in female naïve versus primed cells – is X_i_ reactivation incomplete in the naïve cells, or are they in an early stage of XCI?

In conclusion, our study takes a step further in understanding the dynamics of the X chromosome during human development and the naïve state of human PSCs (Fig. 6). The recent capability of cultivating human embryos *in vitro* until day 13^23, 24^ will allow the investigation of the subsequent development of dosage compensation in the X chromosome beyond the blastocyst stage.

## Methods

### Data acquisition

Dataset-1 containing 1,529 single-cell RNA-seq (scRNA-seq) from 88 human embryos was acquired at ArrayExpress – functional genomics data (EMBL-EBI - http://www.ebi.ac.uk/) under the accession number E-MTAB-3929^7^. The data had been previously filtered by the authors to exclude outlier cells based on gene expression^7^. For the present study we considered embryos with at least four cells sequenced. These data comprise embryos described as follows: 8-cell stage (E3: N = 12, n = 78), morula stage (E4: N = 14, n = 187), fifty-eight blastocysts from embryonic day E5 (N = 23, n = 375), E6 (N = 18, n = 415) and E7 (N = 17, n = 465); where N is the number of embryos and n the number of cells.

Dataset-2 including scRNA-seq from human oocytes, preimplantation embryos and male human embryonic stem cells were acquired at GEO under the accession number GSE36552 (Gene Expression Omnibus - https://www.ncbi.nlm.nih.gov/gds)^8^. Specifically: three oocytes, three zygotes, three 2-cell stage embryos (two cells each), three 4-cell stage (four cells each), three 8-cell stage (four cells from embryo #1 and eight cells from embryos #2 and #3), two morula stage (eight cells each), three blastocyst stage (twelve cells from embryo #1, ten from embryo #2 and eight from embryo #3) and one human embryonic stem cell line at passage zero (with seven sequenced cells). These data had also been previously screened by the authors and they found no outlier cells^8^.

RNA-seq data of two inner cell masses (ICM) and trophectoderm (TE) from one natural monozygotic twin embryo at blastocyst stage were obtained at SRA, under the accession number SRP063754 (Sequence Read Archive - http://www.ncbi.nlm.nih.gov/sra)^10^.

Data from 15 scRNA-seq human fibroblasts (T2N, human fetal primary fibroblast culture derived from female post-mortem skin tissue fetus) were available on the European Genome-phenome Archive (https://www.ebi.ac.uk/ega/datasets) under the accession number EGAD00001001084^9^.

RNA-seq of the female hES cell line H9 were acquired at ArrayExpress – functional genomics data (EMBL-EBI - http://www.ebi.ac.uk/) under the accession number E-MTAB-2857^11^.

### Data processing

To filter out low-quality reads, we used Trimmomatic v0.33^25^ with the default parameters. Briefly, the program was set to remove bases from each read’s ends if presenting sequence quality below 3. The average quality score was calculated using a sliding window of 4 bp. If the score was equal or less than 15, the four successive nucleotides were discarded from the read. Our parameters were also set to exclude reads shorter than 36 bp.

### Reads alignment and counting

Reads were aligned to human assembly hg19 with the respective UCSC genome annotation (obtained from UCSC, GCA000001405.1 at http://hgdownload.cse.ucsc.edu) with TopHat (v2.0.13)^26^. Only uniquely aligned reads were considered for further analyses. Aligned reads were then processed with SAMtools^27^ and the number of reads mapping to each gene was counted using HTSeq^28^.

### Expression analyses

The resulting count files for each cell/sample were used to calculate rpkm (reads per kilobase per million)^29^ and tpm (transcripts per million)^30^. As defined by Yan *et al*.^8^, only genes presenting rpkm ≥0.1 were considered as expressed. As proposed by Wagner *et al*.^30^, tpm values were used in order to compare expression levels among different samples. Outlier cells were removed from all expression analyses to increase robustness.

### Sexing

To determine the sex of each embryo from dataset-2^8^, we considered Y-linked expressed genes (rpkm ≥0.1) outside of the pseudoautosomal region. The total number of Y-expressed genes per embryo was counted and compared to those found in all oocytes and in the male embryonic stem cell line (hESp0) with Fisher’s exact test and Bonferroni’s correction for 18 tests (corrected *P*-value ≤ 0.00277). As the main embryonic genome activation (EGA) occurs at the 8-cell stage^8, 12, 13^, embryos prior to this phase were not considered for sexing. We expected female embryos to show significantly lower numbers of Y-linked expressed genes than the male stem cell line but equal to oocytes. Analogously, male embryos should present significantly more Y-linked expressed genes than oocytes but not different than the male stem cell line (Supplementary Table S1).

For dataset-1, we considered sexing as reported by the authors^7^. However, we identified one female E3 embryo with low number of X-linked SNPs (embryo E3.1: mean = 2.8; female group mean = 12.44, Wilcoxon non-paired *P*-value = 0.0178), and one male E6 embryo with high number of X-linked SNPs (embryo E6.18: mean = 2.739; male group mean = 0.3885; Wilcoxon non-paired *P*-value = 0.0005), resulting in inconsistent sexing. Those embryos were excluded from our analyses.

### Ploidy

Post-EGA embryos had their cell ploidy analyzed using methods described by Mayshar *et al*.^31^. The male human embryonic stem cell line at passage zero (hESp0, dataset-2), karyotyped as 46,XY, was used as a normal control^8^. For each cell from a particular embryo, individual gene expression values (tpm) were normalized by its median value across all embryo cells. In order to obtain a visual profile of each chromosome in different embryos, the normalized gene expression values were then used to build a moving average plot (window size 188 bp). Embryos showing visual abnormalities for a particular chromosome were checked for an excess of over or underexpressed genes using Chi-square test. Genes expressed in one cell by more than 1.5 or less than 0.67 fold in comparison to the embryo median value were considered over/underexpressed, respectively.

### Variant calling and SNP annotation

We used VarScan (v2.3.7, http://varscan.sourceforge.net)^32, 33^ for variant calling with the option mpileup2cns to retrieve reference and alternative allelic read counts covering single nucleotide variants (SNV). SNVs were then annotated using dbSNP Build 138 (GRCh37.p10). Ambiguous dbSNP identification numbers (IDs), *i.e*., SNPs with the same ID annotated in different positions on the genome, were removed prior to annotation. IDs mapped into intergenic regions, pseudogenes, or regions containing overlapping genes were also removed in order to avoid false biallelic detection.

### Embryo genotyping and detection of monoallelic and biallelic expressed genes

The relative expression ratio of two different alleles was calculated by directly counting the allele-specific reads covering a SNP position mapped to the reference or the alternative allele and then dividing it for all reads covering that position. Only SNPs position covered by at least 20 reads were considered. Biallelic expression was considered when allele relative expression ratio ranged from 0.2 to 0.8; and monoallelic expression otherwise^9^. The same SNP should be detected in at least two cells of the same embryo or cell line to be considered in the downstream analyses. We also excluded, from all samples, X-linked SNPs showing biallelic expression in the male hESp0 stem cell line.

To consider a gene as heterozygous (informative), it should meet one of the following criteria: (i) to contain SNPs showing biallelic expression or (ii) to have at least one SNP showing monoallelic expression, *i.e*., different cells from the same embryo or cell line expressing different alleles. However, if a gene with two or more SNPs identified in the same cell displayed both (i) and (ii), the inconsistent gene was removed from that cell’s analyses.

The analyses presented in Petropoulos *et al*.^7^ used a minimum of 3 reads for a valid SNP call and considered as biallelic those SNPs with allele relative expression ratio ranging from 0.1 to 0.9. Since the number of reads covering a SNP position influences the rate of false positive and negative biallelic calls, we used more stringent parameters, considering only SNPs position covered by at least 20 reads, and excluding inconsistent genes as described above. Dataset-1 showed low number of reads aligned to genes when compared to other scRNAseq sets (dataset-2 and human fibroblasts^9^) (Supplementary Fig. S2). Therefore, the number of annotated SNPs and heterozygous genes were also significantly lower (Supplementary Fig. S2).

We deposited the script used in our analyses in the following link: https://github.com/gustribeiro/Mello_analysis_pipeline.

### Rates of monoallelic and biallelic expressed genes during development

In female embryos, biallelically expressed genes on the X chromosome indicate that both Xs are active, and therefore an increase in the fraction of heterozygous monoallelically expressed genes is expected when random XCI takes place. In contrast, due to the presence of maternal RNAs, we expected very early male embryos to show biallelic expression of X-linked genes that decreases during development as maternal mRNA resources extinguish^7, 8^. Later in development, no heterozygous X-linked genes would be expected, as males are hemizygotes (see Supplementary Fig. S1 for a scheme of the model).

In order to test the correlation between developmental stage and the fractions of biallelic and monoallelic expression, we first assessed for each cell the numbers of (i) all expressed genes, (ii) biallelic and (iii) monoallelic heterozygous genes. To calculate the fractions of biallelically and monoallelically expressed genes, the number of biallelic genes as well as the monoallelic genes, respectively, was divided by the total number of expressed genes for each cell at each stage. Genes located in the pseudoautosomal region were excluded from the analyses.

Significant increase and decrease in fraction of monoallelically or biallelically expressed genes were assessed by testing respectively positive and negative correlations among those measures and the developmental stages with Pearson correlation coefficient. For comparisons of only two developmental stages, we used non-paired Wilcoxon test.

To avoid allelic variance derived from the substantial amount of maternal mRNA^8^, we excluded 2- and 4-cell stage embryos from the biallelic correlation analyses in dataset-2. Since two maternal Xs are active at the beginning of embryo development (see X:A expression ratio analyses), pre-EGA embryos should be the starting point for measuring the increase of monoallelic expression. We noted that the number of embryo cells analyzed could increase the chances of detecting monoallelic expression in heterozygous genes. Therefore, we randomly picked four cells of each embryo (4-cell, 8-cell, morula, blastocyst and hESp0) for statistical correlation analyses in Fig. 1. Males and females were plotted and tested separately for X chromosome analyses, while all embryos were pooled together for analyses of autosomal genes. Similar results were found when considering all cells of each embryo for statistical correlation analyses of monoallelically X-linked expressed genes and using lower numbers of genes, measuring correlations using individual autosomes (Supplementary Fig. S1 and Supplementary Fig. S4).

### Expression levels of X-linked biallelically expressed genes

Expression levels of X-linked biallelically expressed genes (with rpkm ≥ 0.1) were taken from each cell of female embryos. Transcripts per million (tpm) sum and median were then calculated for each individual cell and grouped by developmental stage (8-cell, morula and blastocyst for each dataset). Differences between the groups were tested with non-paired Wilcoxon test.

For the gene-wise analysis of female-to-male expression ratios, we calculated for each X-linked biallelically expressed gene in females the mean expression in female and male cells at each developmental stage ($\bar{{\rm{X}}}$_f_ and $\bar{{\rm{X}}}$_m_, respectively). After excluding those with a mean expression <5 rpkm, we calculated the female-to-male mean expression ratio ($\bar{{\rm{X}}}$_f_/$\bar{{\rm{X}}}$_m_) for each gene in each developmental stage and then plotted the distribution. The distributions between stages were compared with paired Wilcoxon test. In order to avoid distortions of few very high or very low expressed genes, outliers were removed prior to plots and tests.

### Allelic frequency on human X chromosome and the *XIST* gene

Allelic frequencies in the X chromosome and the *XIST* gene were obtained from data generated by the 1000 Genomes Project phase 3 (http://www.1000genomes.org/)^34^. We considered global allelic frequencies in females of all known SNPs on the *XIST* gene and those along the entire X chromosome excluding pseudoautosomal region. The differences were assessed using non-paired Wilcoxon test.

### X chromosome to autosomes expression ratio

For each individual cell, median gene expressions (tpm) were calculated for all expressed autosomal and X-linked genes outside of pseudoautosomal region. X:A ratios were obtained for individual cells of each embryo by dividing the median tpm values of the X chromosome by those of the autosomes. For dataset-2^8^ non-paired Wilcoxon tests were used to assess differences between pre-EGA samples (oocytes, zygotes, 2-cell and 4-cell stage) and the following stages (8-cell, morula and blastocyst,) male and female hESCs, and female fibroblast cell line; and between male and female embryos at the 8-cell and blastocyst stages.

### Statistical tests

All statistical tests were performed using R version 3.1.3 (http://www.R-project.org/)^35^.

## Electronic supplementary material

Supplementary Material

Supplementary Table S1

Supplementary Table S2

Supplementary Table S3

Supplementary Table S4
