# Supplementary material for: Early X chromosome inactivation during human preimplantation development revealed by single-cell RNA-sequencing
Source: Sci Rep. 2017 Sep 7;7:10794. doi: 10.1038/s41598-017-11044-z (PMC5589911; doi:10.1038/s41598-017-11044-z)
Supplement: Supplementary file 1 — Supplementary Material [file 41598_2017_11044_MOESM1_ESM.pdf]

Early X chromosome inactivation during human preimplantation development revealed by single-cell RNA-sequencing.

Joana C. Moreira de Mello, Gustavo R. Fernandes, Maria D. Vibranovski, Lygia V. Pereira.

SUPPLEMENTARY MATERIAL

Supplementary Figure S1.

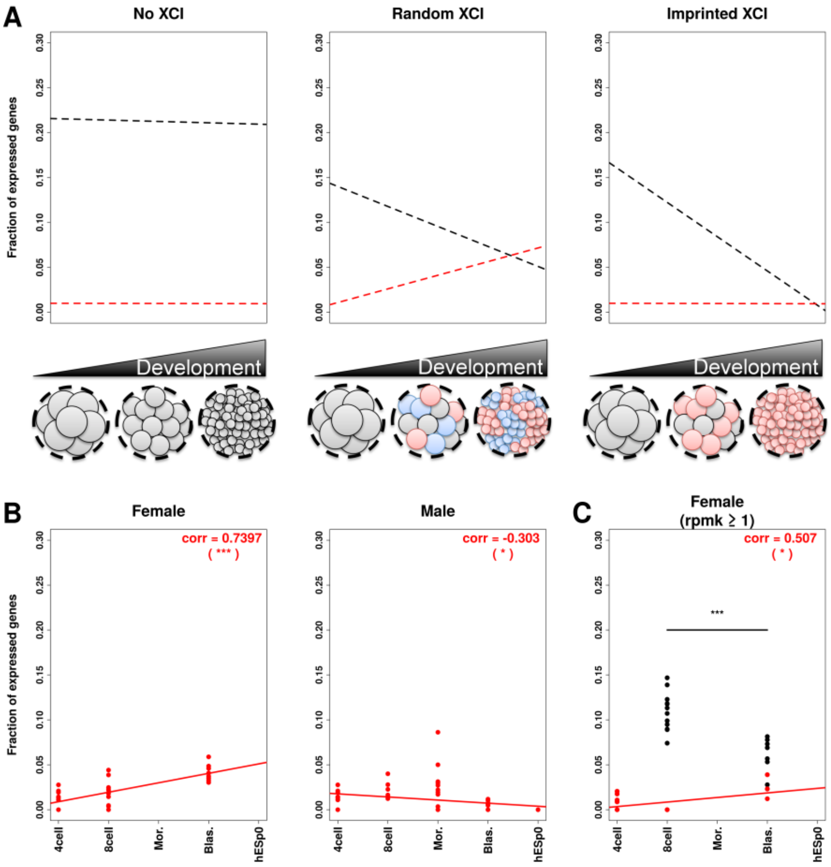

Supplementary Figure S1. Analyses of genes with mono and biallelic expression during development. (A) Schematic model of expected allele-specific expression patterns in embryos with no XCI, random XCI and imprinted XCI during preimplantation development. Cells with biallelic and monoallelic gene expression are coloured gray and pink/blue, respectively. Left panel: if no XCI takes place, the fraction of biallelically expressed genes (black dashed line in all panels) would not decrease and of monoallelically expressed genes (red dashed line in all panels) would not increase and thus, no significant correlation with the developmental stage should be observed. Below, both X chromosomes are active in all cells of the embryos (biallelic expression, gray cells). Middle panel: in random XCI, we would expect a significant negative correlation for the fraction of biallelically expressed genes as well as a significant positive correlation for the fraction of monoallelic genes with developmental stage. Cells in pink and in blue in the embryos below indicate inactivation of paternal and maternal X, respectively. Right panel: in imprinted XCI, female embryos should present the same pattern as males: a significant negative correlation for biallelically expressed genes and no correlation of monoallelically expressed genes and stages of development. Cells in pink indicate that the entire embryo has the paternal X inactivated. (B) Analysis of X-linked monoallelic expression during preimplantation development in all cells of dataset-2. Fraction of monoallelically expressed genes per total of expressed genes (red) for each sequenced cell. Similar analysis of biallelically expressed genes is shown in Fig. 1B. Pearson's r and *P*-values are depicted in each panel. (C) Female X-linked genes from dataset-2 with rpkm ≥ 1.0. Fractions of genes with biallelic (black) and monoallelic (red) expression per total of expressed genes are shown for each analyzed cell.

Pearson's r and *P*-values are depicted in red for monoallelic expressed genes; non-paired Wilcoxon test in black were performed to compare differences in biallelic expressed genes between two stages. *P*-value (*) < 0.05, (***) ≤ 0.001.

Supplementary Figure S2.

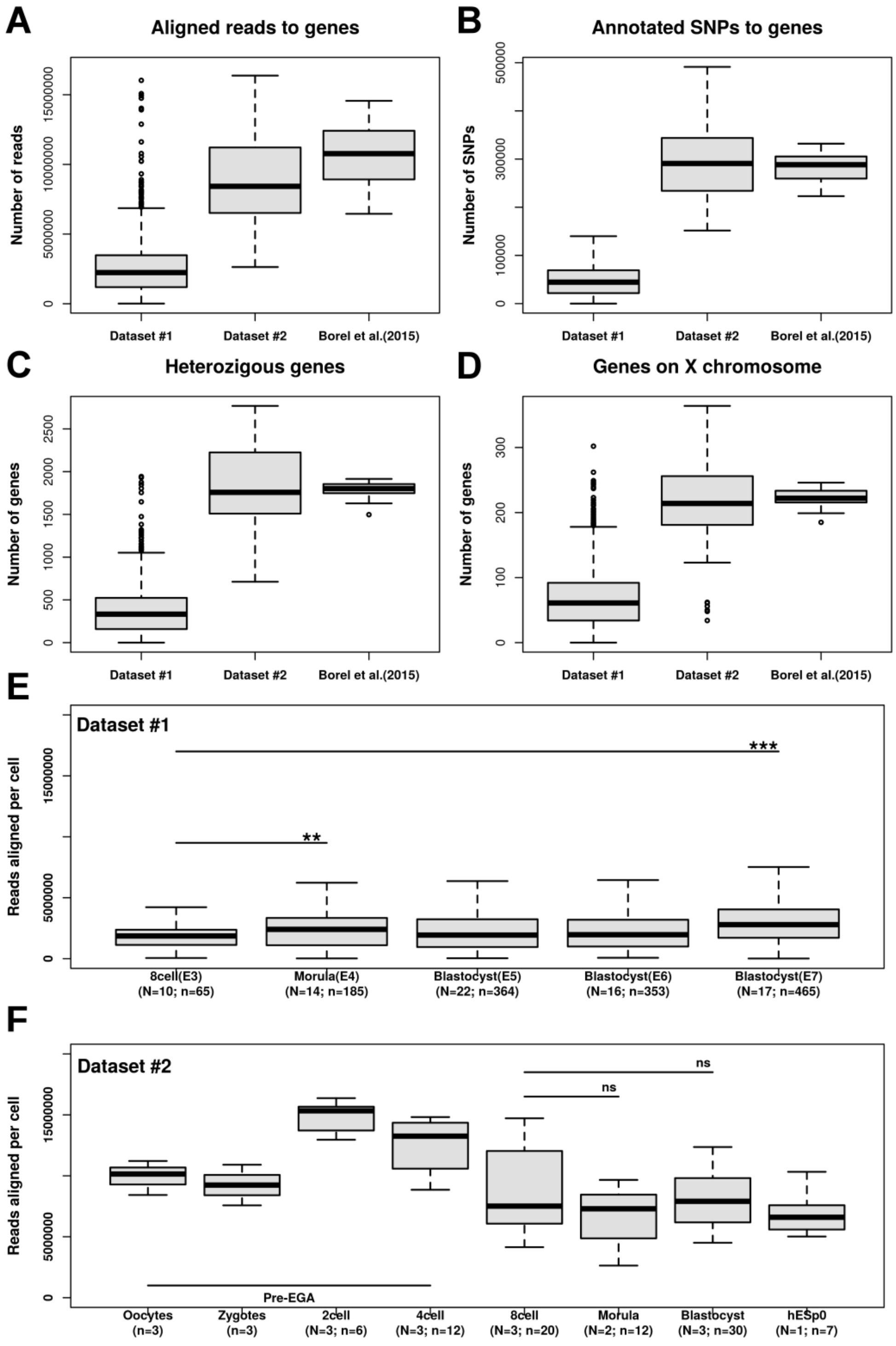

Supplementary Figure S2. Comparisons of scRNA-seq data. Borel et al. (2015) refers to scRNAseq dataset from female adult fibroblasts[9]. (A) Box-plots for number of reads aligned to genes. (B) Box-plots for number of annotated SNPs with read coverage ≥ 20. (C) Box-plots for number of heterozygous genes found for annotated SNPs in (B). (D) Box-plots for the total number of genes on the X chromosome found for annotated SNPs in (B). The same methods and parameters were used to analyse the three studies. The distributions of numbers of aligned reads in each cell of each developmental stage are show for (E) dataset-1 and (F) dataset-2. Number of embryos (N) and number of cells (n) are indicated for each stage. Non-paired Wilcoxon test. *P*-value (**) ≤ 0.01; (***) ≤ 0.001; (ns) not significant. Although the total numbers of sequenced reads differ between the two datasets, they share nearly 85% of the expressed genes with rpkm ≥ 0.1 (data not shown).

Supplementary Figure S3

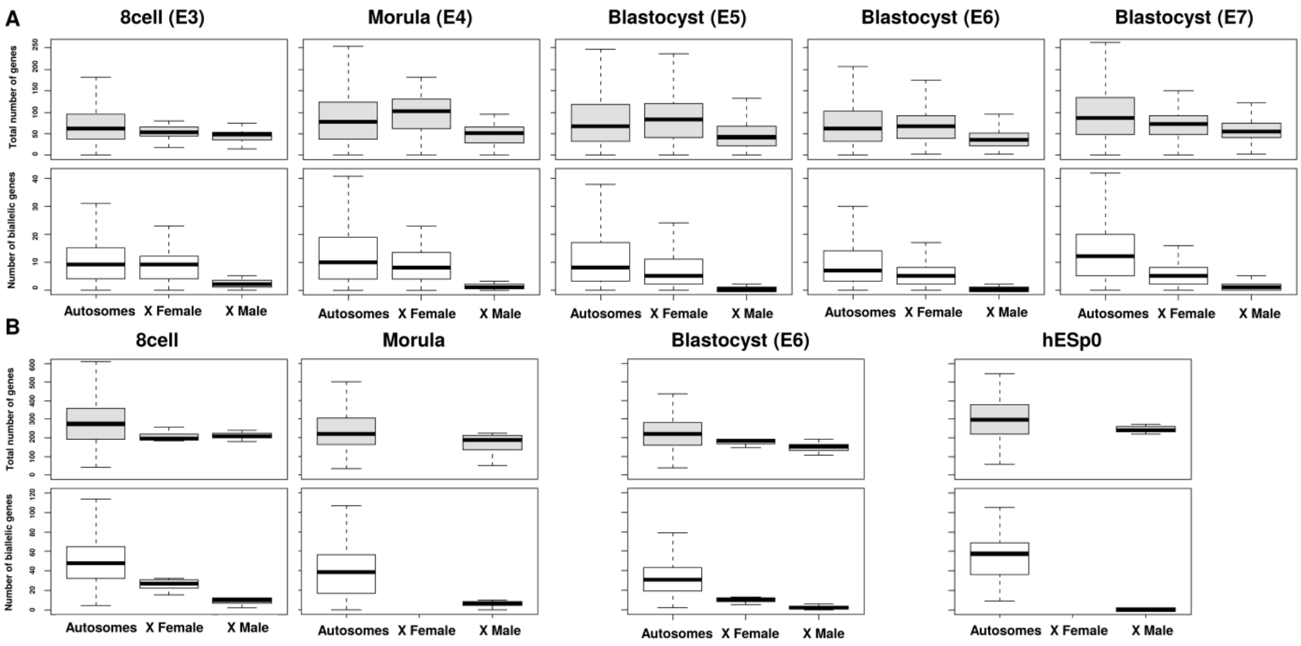

Supplementary Figure S3: Number of genes analyzed per stage. Distributions of the total numbers of genes (gray) and of biallelic genes (white) in autosomes and in the X chromosome in cells from each developmental stage used in the analyzes of allelic expression in Fig. 1 and Supplementary Fig. S1.

Supplementary Figure S4

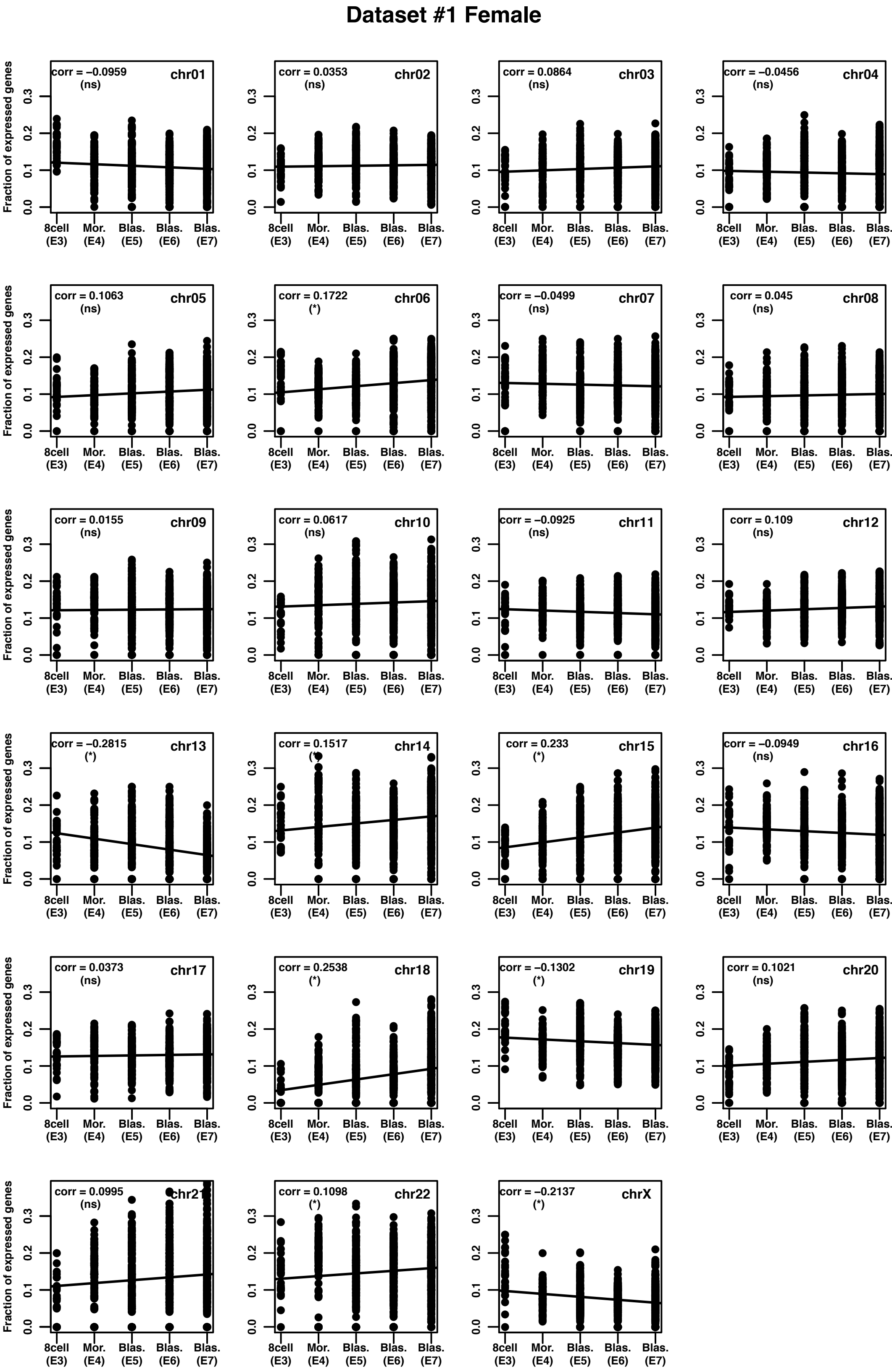

Dataset #1 Female
Fraction of expressed genes
0.0
0.1
0.2
0.3
8cell (E3)
Mor. (E4)
Blas. (E5)
Blas. (E6)
Blas. (E7)
corr = −0.0959 (ns) chr01
corr = 0.0353 (ns) chr02
corr = 0.0864 (ns) chr03
corr = −0.0456 (ns) chr04
corr = 0.1063 (ns) chr05
corr = 0.1722 (*) chr06
corr = −0.0499 (ns) chr07
corr = 0.045 (ns) chr08
corr = 0.0155 (ns) chr09
corr = 0.0617 (ns) chr10
corr = −0.0925 (ns) chr11
corr = 0.109 (ns) chr12
corr = −0.2815 (*) chr13
corr = 0.1517 chr14
corr = 0.233 (*) chr15
corr = −0.0949 (ns) chr16
corr = 0.0373 (ns) chr17
corr = 0.2538 (*) chr18
corr = −0.1302 (*) chr19
corr = 0.1021 (ns) chr20
corr = 0.0995 (ns) chr21
corr = 0.1098 (*) chr22
corr = −0.2137 (*) chrX

Dataset #1 Male

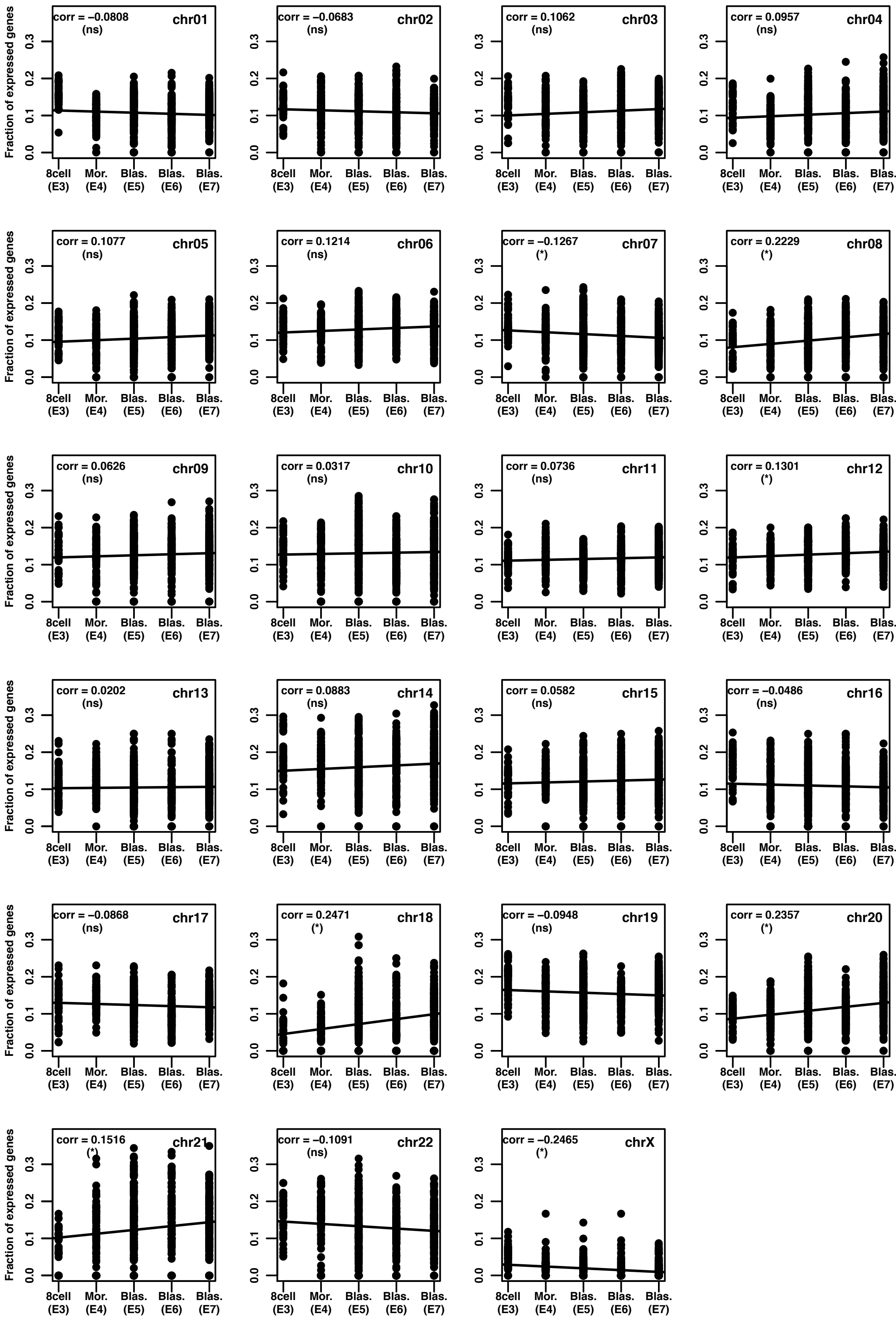

Dataset #2 Female

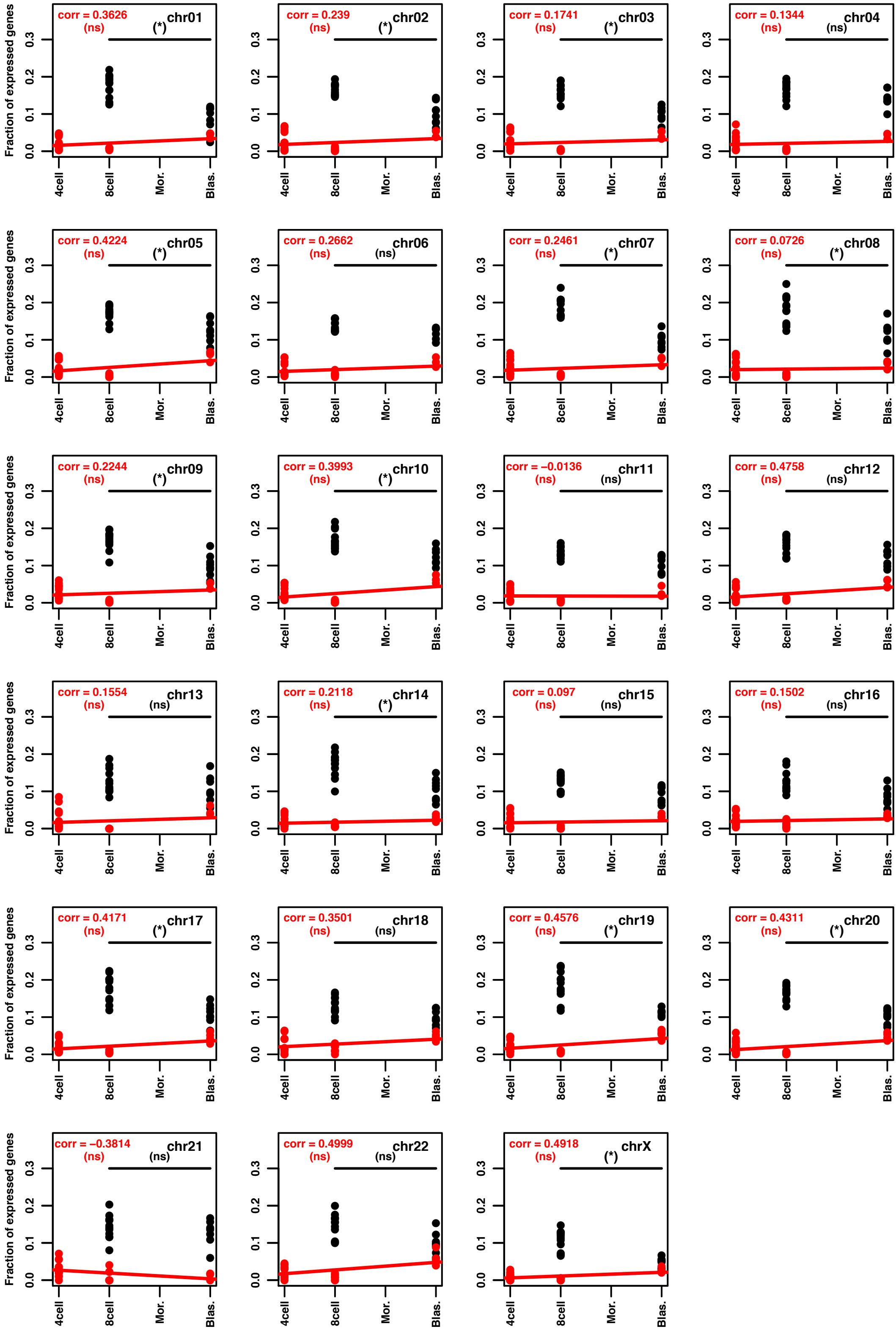

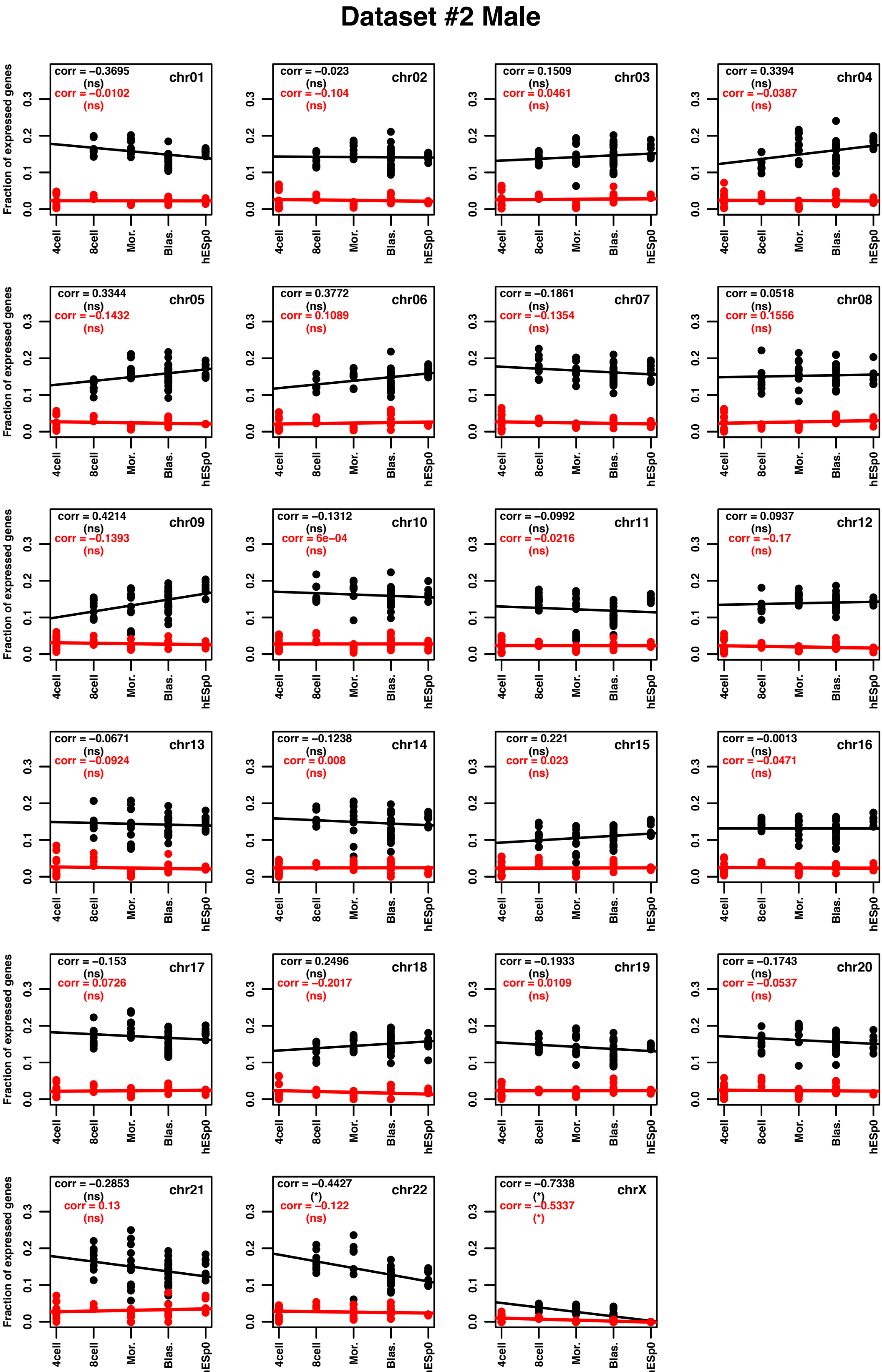
Dataset #2 Male
Fraction of expressed genes
4cell
8cell
Mor.
Blas.
hESp0
corr = −0.3695 (ns) corr = −0.0102 (ns) chr01
corr = −0.023 (ns) corr = −0.104 (ns) chr02
corr = 0.1509 (ns) corr = 0.0461 (ns) chr03
corr = 0.3394 (ns) corr = −0.0387 (ns) chr04
corr = 0.3344 (ns) corr = −0.1432 (ns) chr05
corr = 0.3772 (ns) corr = 0.1089 (ns) chr06
corr = −0.1861 (ns) corr = −0.1354 (ns) chr07
corr = 0.0518 (ns) corr = 0.1556 (ns) chr08
corr = 0.4214 (ns) corr = −0.1393 (ns) chr09
corr = −0.1312 (ns) corr = 6e−04 (ns) chr10
corr = −0.0992 (ns) corr = −0.0216 (ns) chr11
corr = 0.0937 (ns) corr = −0.17 (ns) chr12
corr = −0.0671 (ns) corr = −0.0924 (ns) chr13
corr = −0.1238 (ns) corr = 0.008 (ns) chr14
corr = 0.221 (ns) corr = 0.023 (ns) chr15
corr = −0.0013 (ns) corr = −0.0471 (ns) chr16
corr = −0.153 (ns) corr = 0.0726 (ns) chr17
corr = 0.2496 (ns) corr = −0.2017 (ns) chr18
corr = −0.1933 (ns) corr = 0.0109 (ns) chr19
corr = −0.1743 (ns) corr = −0.0537 (ns) chr20
corr = −0.2853 (ns) corr = 0.13 (ns) chr21
corr = −0.4427 (*) corr = −0.122 (ns) chr22
corr = −0.7338 (*) corr = −0.5337 (*) chrX

Supplementary Figure S4: Allelic expression during preimplantation development in individual chromosomes. Analyses of changes in fractions of biallelically/monoallelically expressed genes during female and male development in preimplantation embryos from datasets -1 and -2. Fractions of biallelically (black) and monoallelically (red) genes per total of expressed genes in each cell for each developmental stage are shown for each individual chromosome. X axis refer to embryonic developmental stage, dataset-1: 8-cell (E3); morula (E4); early, mid and late blastocyst (E5, E6, E7, respectively); dataset-2: 4-cell; 8-cell; morula (Mor.); blastocyst (Blas.), male human ESC line at passage 0 (hESp0). Pearson's r values (corr) are depicted in each panel. Statistical significance threshold for *P*-value was set at 0.00217 which corresponds, according to the Bonferroni's correction, to the 0.05 value divided by 23 chromosomes tested. (*) *P*-values ≤ 0.00217; (ns) not significant.

Supplementary Figure S5

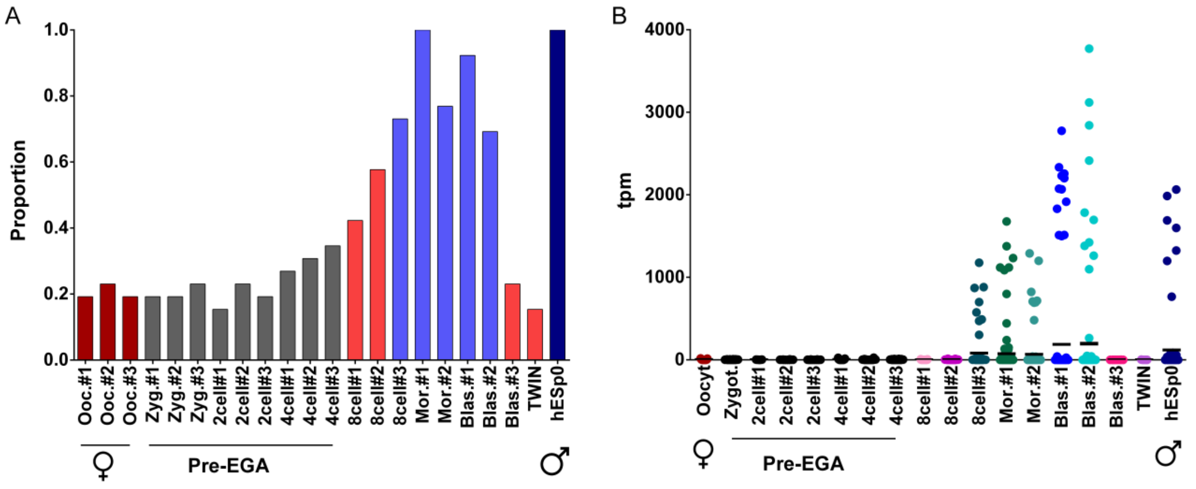

Supplementary Figure S5. Sexing human preimplantation dataset-2 embryos. (A) Proportion of Y-linked genes expressed in each embryo normalized by the number of Y-linked genes expressed in male hESp0 cells. Oocytes in red; hESp0 in dark blue; pre-EGA stages (from zygotes to 4-cell) in gray; females (8-cell #1, 8-cell #2, blastocyst #3 and Twin) in pink and males (8-cell #3, morulas #1 and #2, blastocysts #1 and #2) in blue classified based on Fisher's exact test comparisons to oocytes and hESp0. Bonferroni corrected *P*-values ≤ 0.0027. Refer to Supplementary Table S1 for numbers and IDs of Y-expressed genes and statistical results. (B) Scatter-plot of Y-linked gene expression level in single cells from oocytes to blastocysts and hESp0 pointing that the majority of Y-linked transcripts are detected only in male samples. The mean is shown as a black horizontal line. tpm, transcripts per million.

Supplementary Figure S6.

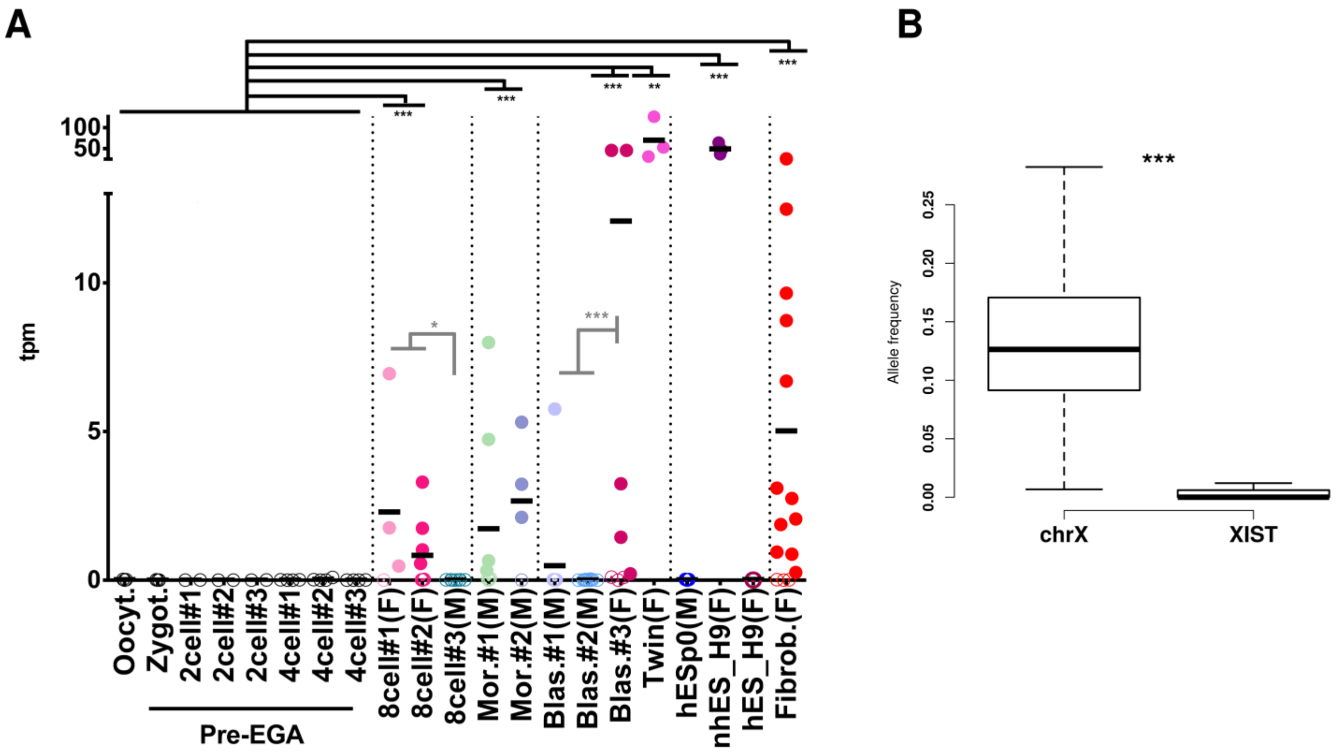

Supplementary Figure S6. *XIST* expression and heterozygosity.

XIST expression during human preimplantation development. (A) Scatter plot of *XIST* expression level in single-cells at different developmental stages from dataset-2. Twin corresponds to ICMs and trophectoderm of a female blastocyst with two ICMs[10]; Fibrob., to a fibroblast female cell line[9]; hES_H9 to a female hESC line and nhES_H9 to its naïve counterpart[11]. Open circles rpkm < 0.1; closed circles rpkm ≥ 0.1; tpm, transcripts per million. (B) Analysis of SNPs in *XIST* gene sequence. Comparison of allelic frequencies of SNPs along the X chromosome exons with those in the *XIST* gene using data from the 1,000 Genomes[34]. The mean allelic frequency found in *XIST* was significantly lower when compared to that for all X-linked genes outside of the

pseudoautosomal region (0.018 and 0.138 respectively). Median values of allelic frequency were 0 and 0.126 for the *XIST* gene and the X chromosome, respectively). *P*-value (*) < 0.05; (**) ≤ 0.01, (***) ≤ 0.001 for non-paired Wilcoxon test.

Supplementary Figure S7.

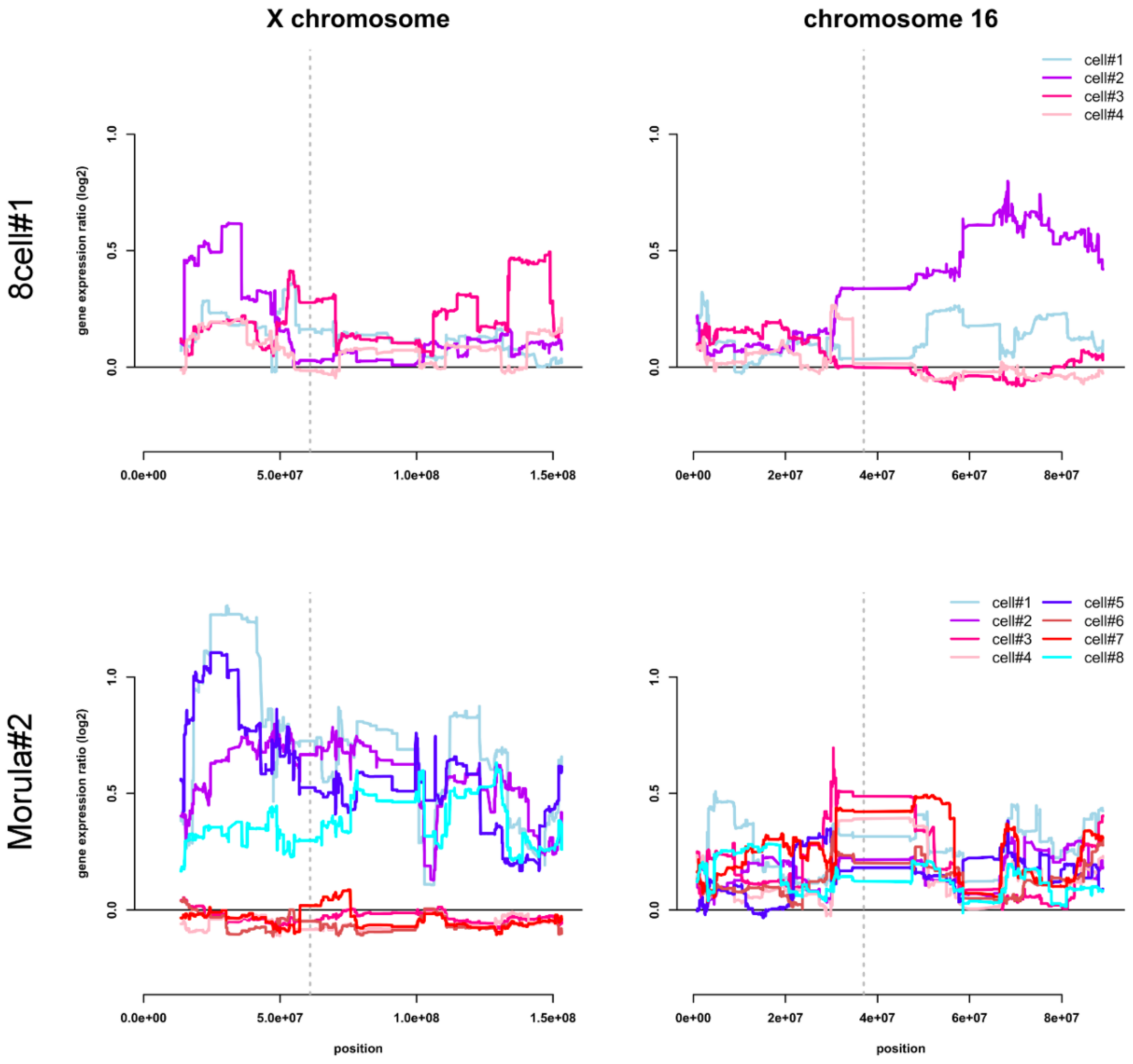

Supplementary Figure S7. Ploidy analyses of single-cells. Moving average plot showing aberrant patterns of ploidy for morula #2 and 8-cell embryo #1 from dataset-2. Morula #2 presented cells #3, #4, #6 and #7 with significant lower expression of X-linked genes, indicating absence of X chromosome. In cell #2 of 8-cell embryo #1, the 16q region showed a significant overexpression in comparison to other cells. For graphic comparison, normal patterns can be observed on chromosome 16 for morula

#2, and X chromosome for 8-cell embryo #1. Dotted lines represent the centromere position. Refer to Supplementary Table S3 for number of over and under expressed genes and statistical results.

Supplementary Material
Moreira de Mello et al. Early X chromosome inactivation during human preimplantation development revealed by single-cell RNA-sequencing.

Supplementary Figure S8

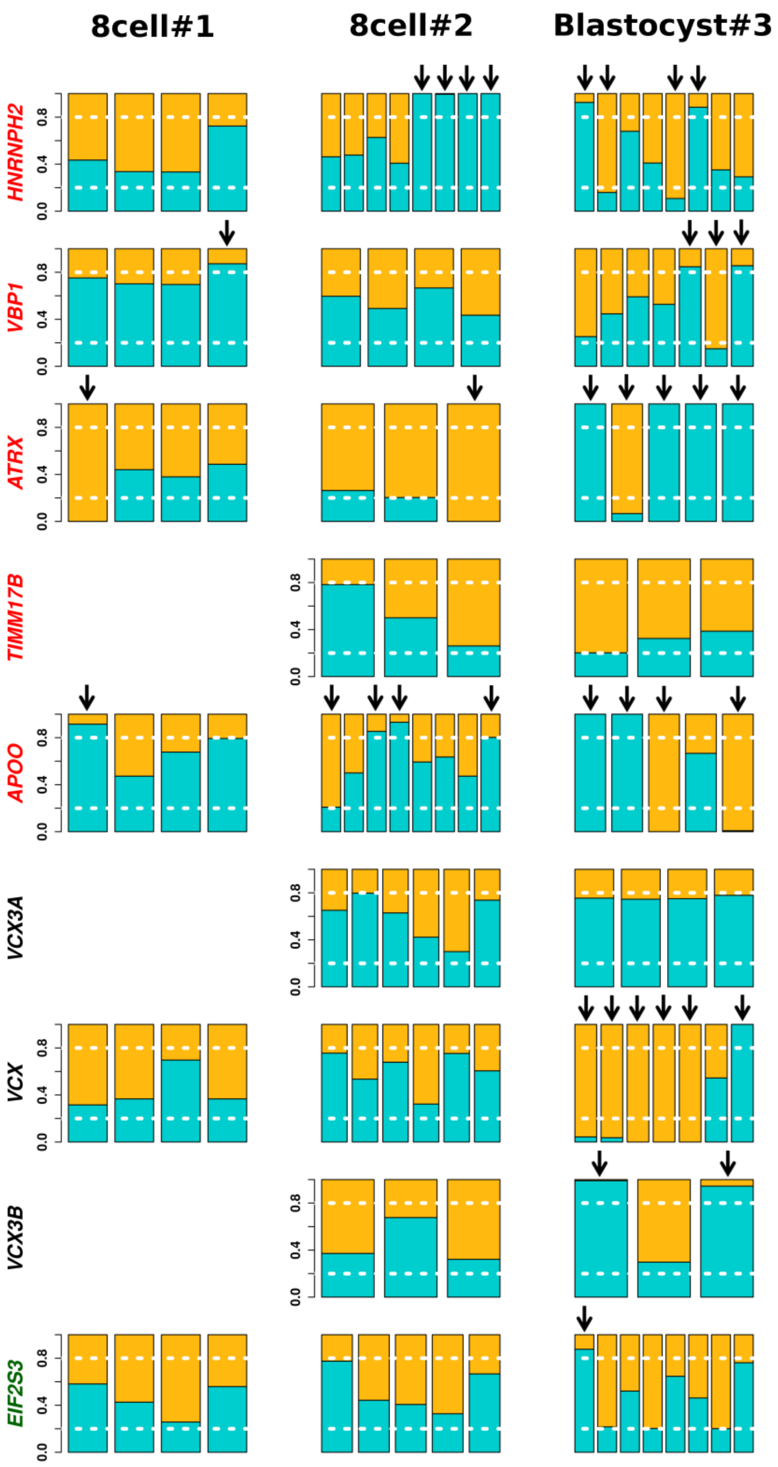

Supplementary Figure S8. Allele-specific expression of X-linked genes at the 8-cell and blastocyst stages. Cells are grouped by embryo where each bar represents a single cell. Allelic expression pattern of informative genes common in female 8-cell embryos #1 and/or #2, and blastocyst #3. Allelic relative expression ratios ≤ 0.2 or ≥ 0.8 were considered as monoallelic expression (white dotted line). Relative expression of reference and alternative alleles in blue and orange, respectively. Arrows point to cells with monoallelic expression. Genes in red are subjected and in green escape XCI, respectively, and those in black are not known[36].

Supplementary Figure S9

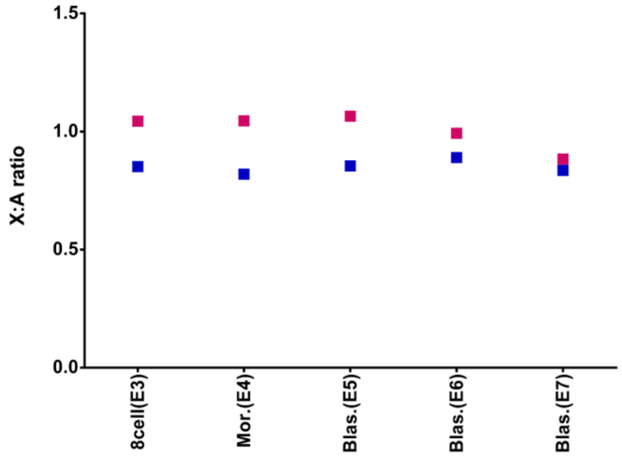

Supplementary Figure S9. Dosage compensation of the X chromosome. Mean X:A expression ratio per stage using dataset-1. Female embryos in pink: 8-cell (n = 5 embryos); morula (n = 7); blastocyst E5, E6 and E7 (n = 10 each). Male embryos in blue: 8-cell (n = 6); morula (n = 7); blastocyst E5 (n = 13); E6 (n = 8); E7 (n = 7).

Supplementary Note

Note S1: *XIST* expression during preimplantation development

As expected, *XIST* was not expressed in male hESp0 (rpkm < 0.1 in all cells) (Supplementary Fig. S4; Supplementary Table S2). In contrast, female fibroblast cells expressed *XIST*, although at variable levels as previously reported for long non-coding RNAs[S1]. Oocytes and pre-EGA embryos had negligible numbers of *XIST* transcripts (rpkm < 0.1), consistent with reactivation of the inactive X (Xi) during oogenesis[16]. At the 8-cell and blastocyst stages, *XIST* expression in female was significantly higher than in pre-EGA or male embryos, corroborating RNA-FISH results of *XIST* activation[4]. Interestingly, both male morulas expressed *XIST* significantly higher than the pre-EGA embryos, as previously observed[5]. In both datasets 1 and 2 we observed a marked increase in *XIST* expression from the morula to the blastocyst stage. Finally, while the line of female primed hESCs (H9) had negligible numbers of *XIST* transcripts, as reported[15], its naïve counterpart (nhESC_H9) expressed *XIST* at levels equivalent to those of female blastocysts (Supplementary Fig. S6; Supplementary Table S2).

No informative SNPs in *XIST* were found in the scRNA-seq dataset-2, initially suggesting monoallelic *XIST* expression in all samples. However, to exclude the possibility of complete *XIST* homozygosity, we compared the allelic frequency of SNPs along the X chromosome with that in *XIST* gene using data from the 1000 Genomes[34]. Interestingly, the mean allelic frequency found in *XIST* was significantly lower when compared to that for all X-linked genes out of the pseudoautosomal region (Supplementary Fig. S6) 0.018 and 0.138, respectively. Non-paired Wilcoxon test:

1540200 *P*-value < 2.2×10^{-16}; median values of allelic frequency were 0.0 and 0.126 for the *XIST* gene and the X chromosome, respectively). Therefore, since we could not exclude the possibility of complete *XIST* homozygosity, we could not distinguish mono from biallelic *XIST* expression. Petropoulos et al.[7] reported one informative SNP in human embryos (dataset-1) indicating biallelic *XIST* expression in a fraction of cells from female blastocysts. Together with our observation of *XIST* expression in male embryos, this indicates that, in contrast to mice, the human maternal *XIST* allele can be expressed in preimplantation embryos, corroborating lack of imprinted XCI in humans[17].

Supplementary reference

S1. Yunusov, D. et al. HIPSTR and thousands of lncRNAs are heterogeneously expressed in human embryos, primordial germ cells and stable cell lines. *Sci Reports.* **6**, 32753 (2016).
